# Japanese encephalitis virus induces human neural stem/progenitor cell death by elevating GRP78, PHB and hnRNPC through ER stress

**DOI:** 10.1038/cddis.2016.394

**Published:** 2017-01-19

**Authors:** Sriparna Mukherjee, Noopur Singh, Nabonita Sengupta, Mahar Fatima, Pankaj Seth, Anita Mahadevan, Susarla Krishna Shankar, Arindam Bhattacharyya, Anirban Basu

**Affiliations:** 1 National Brain Research Centre, Manesar 122051, Haryana, India; 2 Immunology Lab, Department of Zoology, University of Calcutta, 35, Ballygange Circular Road, Kolkata 700019, India; 3 Department of Neuropathology, National Institute of Mental Health & Neurosciences, Bangalore 560029, India

## Abstract

Japanese encephalitis virus (JEV), which is a causative agent of sporadic encephalitis, harbours itself inside the neural stem/progenitor cells. It is a well-known fact that JEV infects neural stem/progenitor cells and decreases their proliferation capacity. With mass spectrometry-based quantitative proteomic study, it is possible to reveal the impact of virus on the stem cells at protein level. Our aim was to perceive the stem cell proteomic response upon viral challenge. We performed a two-dimensional gel electrophoresis-based proteomic study of the human neural stem cells (hNS1 cell line) post JEV infection and found that 13 proteins were differentially expressed. The altered proteome profile of hNS1 cell line revealed sustained endoplasmic reticulum stress, which deteriorated normal cellular activities leading to cell apoptosis. The proteomic changes found in hNS1 cell line were validated *in vivo* in the subventricular zone of JE infected BALB/c mice. Congruent alterations were also witnessed in multipotent neural precursor cells isolated from human foetus and in autopsy samples of human brain clinically diagnosed as cases of JE patients. Endoplasmic reticulum resident chaperone GRP78, mitochondrial protein Prohibitin and heterogeneous nuclear ribonucleoprotein hnRNPC (C1/C2) have been shown to interact with viral RNA. Hence it is proposed that these are the principle candidates governing endoplasmic reticulum stress-induced apoptosis in JEV infection.

Japanese encephalitis virus (JEV), a deadly neurotropic pathogen with single stranded RNA, causes endemic encephalitis in the tropical countries of South-East Asia. Humans of all age group are vulnerable to JEV infection while children below 15 years of age are more prone to it. Nearly 25% of JE cases are reported to be fatal and among survivors nearly half carry several neuropsychiatric disorders,^1^ mainly motor and cognitive deficits. Apart from neurons, the mitotically active neural stem cell pools of brain (sub-ventricular zone and sub-granular zone of hippocampus) are the potential targets of many neurotropic viruses, like Cytomegalovirus (DNA virus), Coxsackievirus, JEV (RNA virus) and Retrovirus such as HIV. JEV particularly has been shown to enter the neural stem progenitor cells (NSPCs) via lipid raft dependent endocytosis^2^ and promote immunological damage in the NSPCs and diminish their proliferation, which accomplish for the neurobehavioural outcome in the survivors.^3, 4^

Proteomics is an emerging analytical tool to discern important cell regulatory proteins and pathways in the host–virus interaction. Proteomic change in the host cell following viral infection provides an insight into viral pathology named as Viral Proteomics.^5^ Identification of altered functional proteins helps in drug design and antiviral approach. Mass spectrometry-based quantitative proteomic study reveals significant information in the field of host–virus interaction such as response of different host cells after Arbovirus infection like Chikungunya,^6^ Dengue^7^ and many more that are under intense research. A previous study from our lab illustrated a broad neuronal proteomic response in JEV infection.^8^ However, till date there is no such report delineating the proteomic alterations in human neural stem cells following JEV infection, which warranted this study.

Here we report the proteomic profile of JEV-infected human neural stem cells (hNS1). Expressions of 13 proteins were found to be significantly altered, revealing endoplasmic reticulum (ER) stress in the stem cells. ER malfunction has been shown to be a causative agent of numerous neurodegenerative, cardiovascular, inflammatory and metabolic disorders. In stressed condition ER tries to safeguard itself by evoking unfolded protein response (UPR), which reinstates ER homeostasis. However, prolonged stress signal leads to cell death.^9, 10^ JEV has been reported to induce UPR in fibroblast and neuronal cells (N18 and NT-2) leading to apoptosis.^11^ We also confirmed the consequences of ER stress in hNS1 cells and found it as an executioner phenomenon leading to stem cell apoptosis. Evidences indicate neural stem cell death in many other cases of viral infection like Zika virus reducing viable number of cortical hNPCs,^12^ Sindbis virus inducing apoptosis in hNPCs^13^ and Cytomegalovirus promoting apoptosis in NSPCs following ER stress.^14^ Zika virus is also reported to infect neural progenitors resulting in microcephaly.^15^ However, clear understandings of death mediators that are governed by ER stress in JEV infection have not yet been established. Our investigations uncovered three viral RNA interacting proteins GRP78, Prohibitin (PHB) and hnRNPC, whose suppression not only reduced viral infection, but also minimized stress response and resultant apoptosis. Hence, we propose these proteins as cardinal modulators of ER stress progressing to apoptosis in human neural stem cells, post JEV infection.

## Results

### Host proteome response post JEV infection in hNS1 cells

**Figure 1:**
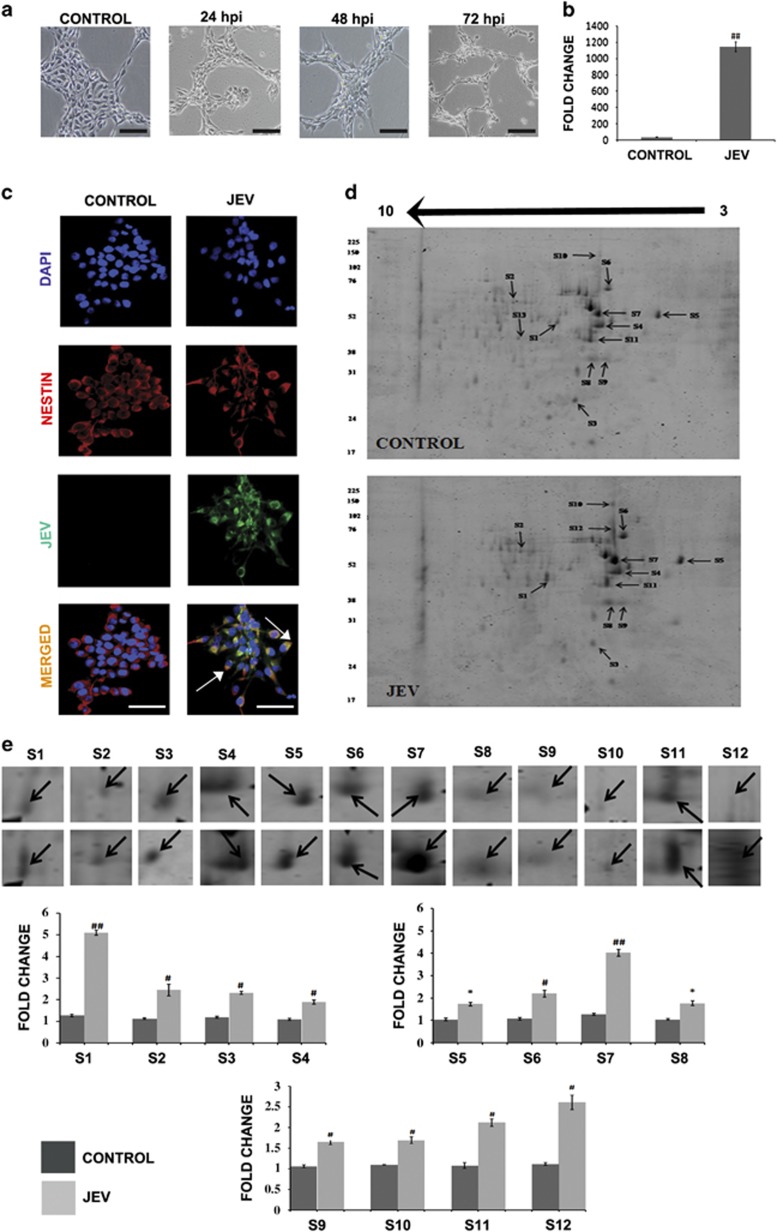
JEV propagates in hNS1 cells mediating proteomic alterations. (**a**) JEV-mediated cytopathic changes were observed in hNS1 cells at different time points of infection (scale bar –50 μm). (**b**) Confirmation of infection was done by real-time PCR at 72 h post infection and significant changes were observed in viral load. (**c**) Immunostaining images also represent JEV infection in all Nestin positive cells (scale bar –20 μm). (**d**) Equal amount of proteins from uninfected and JEV infected hNS1 cells were separated by IPG strips of pH 3–10 (first dimension) and then on 12% SDS-PAGE (second dimension) in which 13 spots were found to be differentially expressed upon viral infection. The spots are labelled in gel image according to Table 1. These spots were marked, excised and identified by MALDI-TOF. (**e**) Bar graphs show relative fold change of the differentially expressed spots (only the spots having fold change ratio |JEV/Control|>=1.5 were taken into consideration). Spot intensities were normalized according to their mean values obtained from duplicate analytical gels after *t*-test analysis (*P<0.05, ^#^*P*<0.01, ^##^*P*<0.001). Data are representative of three independent experiments (mean±S.D.). IPG, immobilized pH gradient

hNS1 cells were first characterized for effective JEV infection. Cytopathic effect post JEV infection was gradually prominent with progressive time point. At 72 h post infection morphological changes were clearly visualized (Figure 1a), which was confirmed by qPCR analysis of JEV genome (Figure 1b) and immunofluorescent staining (Figure 1c). Effective viral replication in hNS1 cells was also confirmed by qRT-PCR and plaque assay experiments of different time points (Supplementary Figures S1A and S1B). Intracellular staining of hNS1 with JEV antigen post 72 h of infection was performed by FACS analysis (Supplementary Figure S1C). Subsequently proteins with altered expressions were identified by proteomic analysis of 72-h JEV infected hNS1 cells comparing with their mock infected controls. Proteins were separated on immobilized pH gradient (IPG) strips at pH range 3–10 on 12% gel and differentially expressed proteins were visualized (Figure 1d). A total of 13 proteins were found to be differentially modulated among which 12 were found to be upregulated (ratio JEV/control >=|1.5|, *P*<0.05) while one spot was absent in infected cells (Figure 1e) when analysed through PD quest software (Bio-Rad, USA). The observed molecular weight values were mostly found to be comparable with the theoretical values of the identified proteins (Table 1).

### Validation studies of differentially regulated proteins in NSPCs

**Figure 2:**
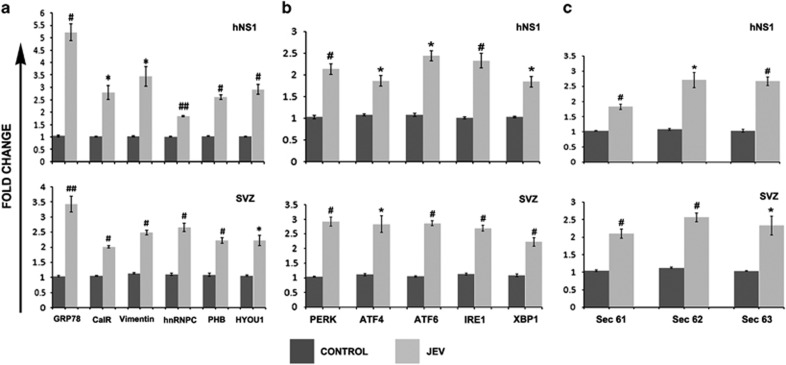
Real-time PCR validation of proteomic data along with studying UPR pathway. (**a**) RNA from hNS1 cells and infected BALB/c mouse SVZ post JEV infection were subjected to cDNA synthesis and qPCR reaction with primers of selective proteins detected in proteomic data. (**b**, **c**) Same cDNA samples were analysed for checking the status of UPR pathway candidate proteins along with Sec complexes. Histograms demonstrate upregulation of the said pathway proteins (*P<0.05, ^#^*P*<0.01, ^##^*P*<0.001). Data are representative of three independent experiments (mean±S.D.). SVZ, sub-ventricular zone

The proteomic results were validated in both hNS1 cells and mitotically active cells in sub-ventricular zone (SVZ) region of mock-infected and JEV-infected BALB/c mice through real-time PCR analysis. SVZ region of BALB/c mice infected with JEV were immunostained and effective viral infection was determined by qRT-PCR experiments (Supplementary Figure S2A and S2B). GRP78, Calreticulin, Vimentin, PHB, hnRNPC and Hyou1 were found to be significantly upregulated post JEV infection (Figure 2a). The data encouraged us to investigate the status of the UPR pathway candidates. Real-time PCR analysis of UPR pathway genes led us to hypothesize that all branches of the established UPR pathway proteins (PERK, IRE-1 and ATF6 and their downstream proteins) are significantly upregulated in JEV infected condition (Figure 2b). Finally we studied the expression profile of Sec complex proteins that are responsible for protein transport pathways in ER. As expected, Sec proteins were found to be significantly expressed in JEV infection, which is probably due to accumulation of large amounts of misfolded proteins to be transported to and from ER (Figure 2c).

### Studying the concomitant consequence of ER stress

**Figure 3:**
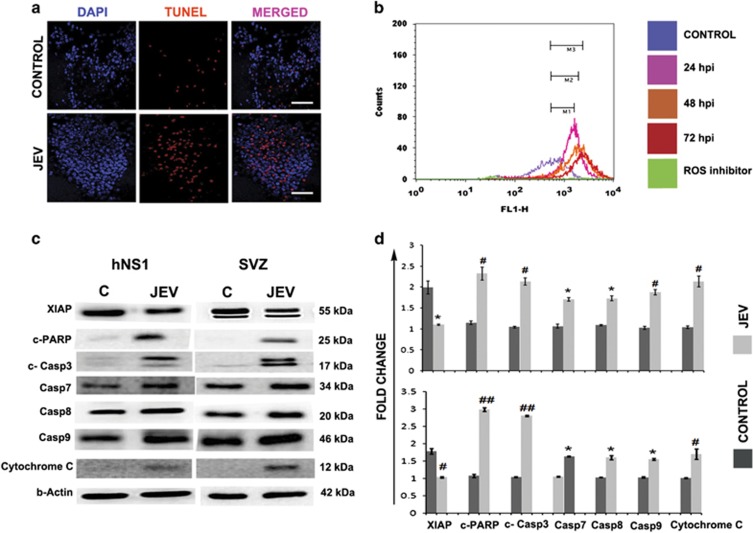
JEV promotes apoptosis in hNS1 cells and mouse SVZ region. (**a**) TUNEL assay was performed to verify the onset of apoptosis in hNS1 cells. Immunocytochemistry data represent more TUNEL-positive cells (indicated by arrows) in JEV infection (scale bar −50 μm). (**b**)More ROS generation was observed post 72 h of infection compared to earlier time points (M3 represents shift in ROS levels post 72 h of JEV infection compared to control, M1 and M2 depicts the same in earlier time points). ROS generation was minimized when cells were pre-treated with 10 μM NAC 2 h prior to JEV infection. (**c**) Prevalence of caspases, c-PARP and cytochrome C post JEV infection is certainly evident in immunoblots of hNS1 cells and BALB/c SVZ samples (one representative *β*-actin blot is shown). (**d**) Bar graph shows the relative fold change in the expression of said proteins both *in vitro* and *in vivo* (*P<0.05, ^#^*P*<0.01, ^##^*P*<0.001). Data are representative of three independent experiments (mean±S.D.). ROS, reactive oxygen species; SVZ, sub-ventricular zone

Signalling through PERK, IRE-1 and ATF6 triggers the pro-apoptotic signals and eventually leads to caspase activation. hNS1 cells were analysed for TUNEL staining post 72 h of JEV infection, which indicated ongoing apoptosis (Figure 3a). reactive oxygen species (ROS) generation, which is an integral part of the phenomenon associated with ER stress, was studied in hNS1 cells. Gradual elevation of ROS levels was observed with progress in viral infection, being highest at 72 h post infection (Figure 3b). Although treating hNS1 cells with ROS inhibitor (NAC) prior to JEV infection neither altered the expression levels of viral NS5 protein nor GRP78, PHB and hnRNPC (Supplementary Figure S3). The presence of apoptotic mediator proteins were validated both in hNS1 cells and 10-day old JEV infected BALB/c mice SVZ region (Figure 3c). Diminished expression of X-linked inhibitor of apoptosis protein and significant elevation of caspases along with notable presence of cytochrome C in NSPCs confirmed the manifestation of apoptosis post JEV infection (Figure 3d). Similar expressions of said proteins were also validated in 3–4-week-old BALB/c mice SVZ region (Supplementary Figure S4).

### JEV RNA interacts with GRP78, PHB and hnRNPC

**Figure 4:**
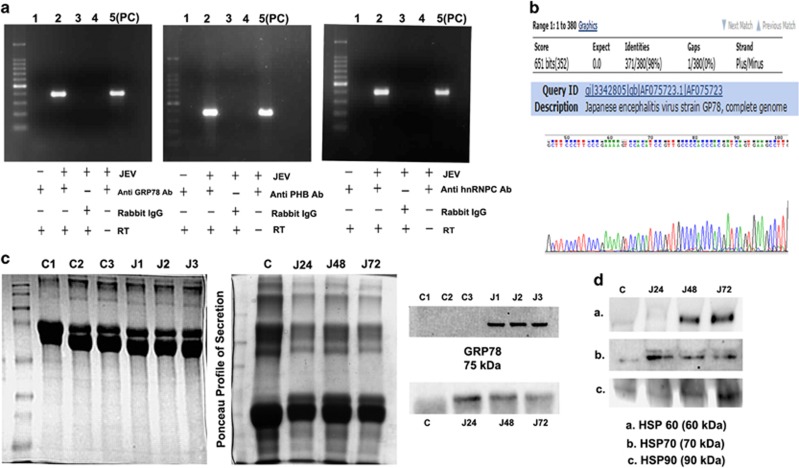
JEV RNA interacts with GRP78, PHB and hnRNPC. (**a**) Viral amplification is visualized only in the samples that were conjugated to GRP78, PHB and hnRNPC antibodies. PC represents positive control of JEV infection. The Co-IP bands appeared in same positions (˜400 bp) with positive control bands implying JEV RNA binding with these proteins. (**b**) Sequencing data (representative image of one of the three Co-IP experiments) reveal more than 95% similarity of the identified band with JEV GP78 genome. (**c**) GRP78 is secreted in hNS1 media post 72 h of infection and later found in progressive time points, ponceau profile of the SDS-PAGE gels indicate equal protein loading in respective experiments. (**d**) Heat shock protein chaperones were also found in hNS1 secretion at progressive time points of JEV infection. (C1, C2, C3 and J1, J2, J3 represent 72 h time point data of three independent experiments; C, J24, J48 and J72 indicate progressive time point data of 24, 48 and 72 h of JEV infection). All data are representative of three independent experiments

RNA co-immunoprecipitation assay was performed in hNS1 cells post 72 h of JEV infection with antibodies against GRP78, PHB and hnRNPC, which revealed the interaction of JEV genomic RNA with these proteins (Figure 4a). No band in IgG and reverse transcription blank panel confirmed specific interaction of JEV RNA with these proteins. Sequencing of the bands post gel extraction, revealed more than 95% similarity with GP78 JEV genome (Figure 4b). Co-localization of the JEV antigen with all three proteins in hNS1 cells further confirmed the interaction (Supplementary Figure S5A, B and C). Studies with other RNA viruses, for example Chandipura virus (CHPV) (Rhabdovirus having a (–) strand RNA), led to the same result. The upregulation of GRP78, PHB and hnRNPC were noted upon CHPV infection in hNS1 cells (24 hpi) and SVZ region of 10-day-old BALB/c mice (Supplementary Figure S6B and C). This clearly led us to hypothesize that these proteins communicate with RNA viruses facilitating their replication inside host NSPCs.

### GRP78 and heat shock chaperone proteins are released from hNS1 cells post JEV infection

GRP78 was found to be secreted into culture media by hNS1 following JEV infection. Proteins were precipitated by TCA followed by immunoblot analysis. Immunoblot with secreted proteins from three independent experiments confirmed the release of GRP78 in JEV infection (Figure 4c). Analysis of time-dependent secretion of GRP78 at 24, 48 and 72 h of JEV infection revealed uniform expression at all time points (Figure 4c). Additionally, important chaperone proteins namely HSP60, HSP70 and HSP90 were found to be present in hNS1 secretome post JEV infection (Figure 4d).

### Ablation of GRP78, PHB and hnRNPC in hNS1 decreases viral load and caspase 3 activation

**Figure 5:**
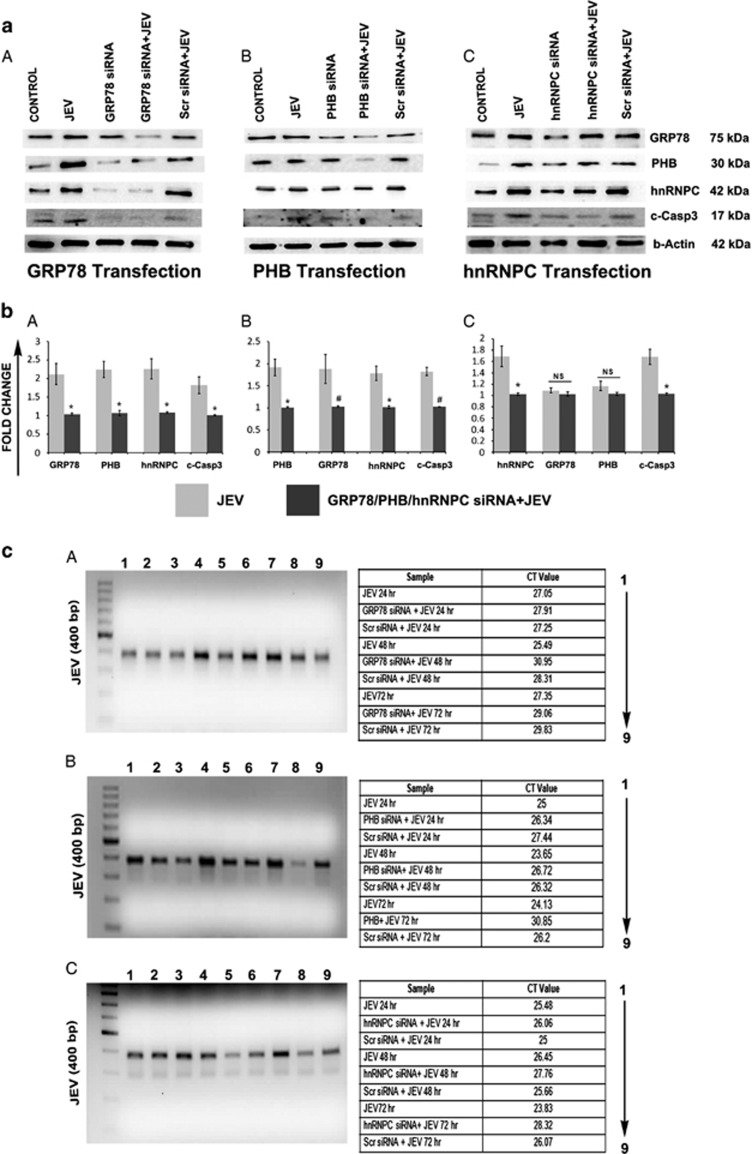
siRNA knockdown of GRP78, PHB and hnRNPC affects mature virion release. (**a**, **b**) siRNA treatment of GRP78, PHB and hnRNPC before viral intervention reduces caspase 3 activation in hNS1 cells. (**a**A, **b**A) Immunoblots show reduced PHB and hnRNPC expression following GRP78 ablation. (**a**B, **b**B) Reduced expression of GRP78 and hnRNPC were observed in case of PHB ablation. (**a**C, **b**C) No significant changes in GRP78 and PHB expression were visualized in case of hnRNPC knockdown. (**c**) In all ablation studies, comprehensible depletion in mature virion release was observed (viral band detected at 400 bp, 1 kb DNA ladder was used) (*P<0.05, ^#^*P*<0.01). Data are representative of three independent experiments (mean±S.D.)

Immunoblot analysis showed decreased PHB, hnRNPC and cleaved caspase 3 expression upon GRP78 ablation by siRNA treatment in hNS1 cells (Figure 5aA, bA). Similar observation was perceived in case of PHB knockdown (Figure 5aB and bB). Interestingly, in case of hnRNPC ablation, only cleaved caspase 3 was found to be notably downregulated (Figure 5aC and bC). Release of JEV virion in cell culture supernatant was then checked by real-time PCR quantitation of the viral RNA. In all three cases of siRNA ablation, released viral particles were found to be remarkably decreased, which is evidenced from respective CT values (Figure 5c). Plaque assay experiment with the 72-h culture supernatant samples showed marked decrease in virion release in all three cases of GRP78, PHB and hnRNPC ablation (Supplementary Figure S7).

### Induction of different cytokines in NSPCs due to presence of JEV

Previous studies have reported the induction of pro-inflammatory cytokines in mouse NSPCs, which led us to hypothesize the production of various cytokines in both JEV-infected SVZ and hNS1 cells. To test the hypothesis, protein samples were prepared from mock-infected and JEV-infected SVZ regions. 30 μg of protein samples were used for Cytokine Bead Array. We found a significant elevation of IL-12, TNF-*α*, IFN-*γ*, MCP-1 and IL-6 in infected SVZ with MCP-1 and IL-6 being most abundant (Supplementary Figure S8A). Significant elevation of IL-1*β*, IL-6 and IL-8 were observed in JEV-infected hNS1 cell culture supernatants (Supplementary Figure S8B). This inflammatory milieu is thought to create a feed forward loop generating more ER malfunction.

### hNS1 proteome response correlates to response of neural progenitors isolated from human foetus

**Figure 6:**
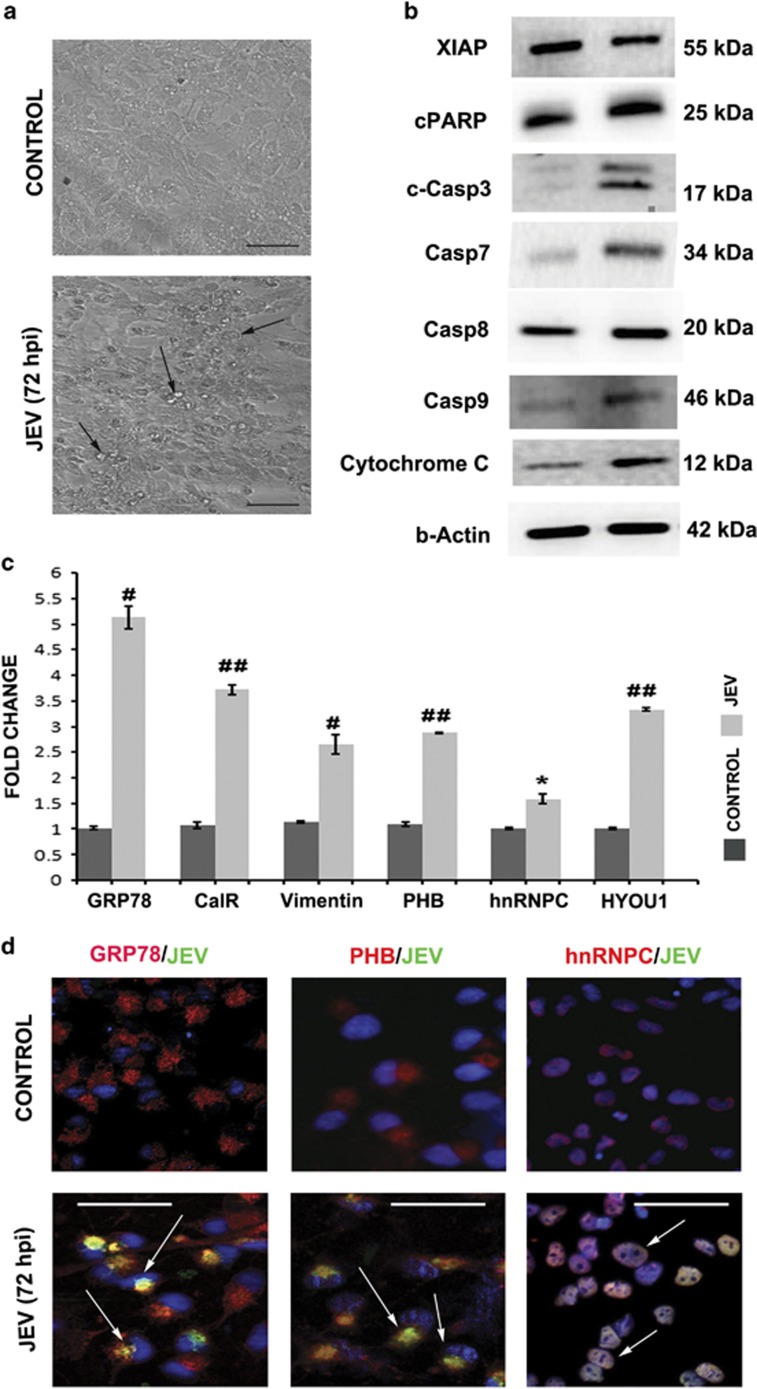
JEV infection promotes ER stress and caspase activation in human neural progenitor cells (hNPCs). (**a**) Multipotent progenitor cells isolated from human foetus were infected with JEV for 72 h. Cells exhibit cytopathic effects post infection (scale bar −50 μm). (**b**) The cells exhibited elevated caspase expression along with cytochrome C release manifesting the onset of apoptosis in hNPCs (densitometry data not shown). (**c**) Histogram of q-PCR represents relative fold change of detected proteins (mass spectrometry) in hNPCs. (**d**) Co-localization of GRP78, PHB and hnRNPC were visualized in these cells post 72 h of JEV infection (scale bar −20 μm) (complete ICC image is given in Supplementary Figure S9) (*P<0.05, ^#^*P*<0.01, ^##^*P*<0.001). Data are representative of three independent experiments (mean±S.D.)

Since hNS1 cells are used as *in vitro* model system to study NSPC proteome post JEV infection, we tried to affirm our findings in primary cultures of hNPCs isolated from human foetus. At first these cells were infected with JEV at MOI of 5 and cytopathic effects were monitored till 72 h (Figure 6a). We validated the presence of proteins detected in mass spectrometry in hNPCs post JEV infection and our previous observation complemented with hNPC data. GRP78, PHB and hnRNPC were significantly elevated in these cells (Figure 6c). We also found the significant increase of caspases along with cytochrome C release (Figure 6b), which reaffirmed about elicitation of similar host response of NSPCs post JEV infection. Immunocytochemistry experiment with the shortlisted proteins that are, GRP78, PHB, hnRNPC, evoking ER stress and apoptosis, distinctly demonstrated co-localization with JEV antigen in hNPCs (Figure 6d,Supplementary Figure S9A, B and C).

### Validation studies in autopsy samples of human encephalitis patients

**Figure 7:**
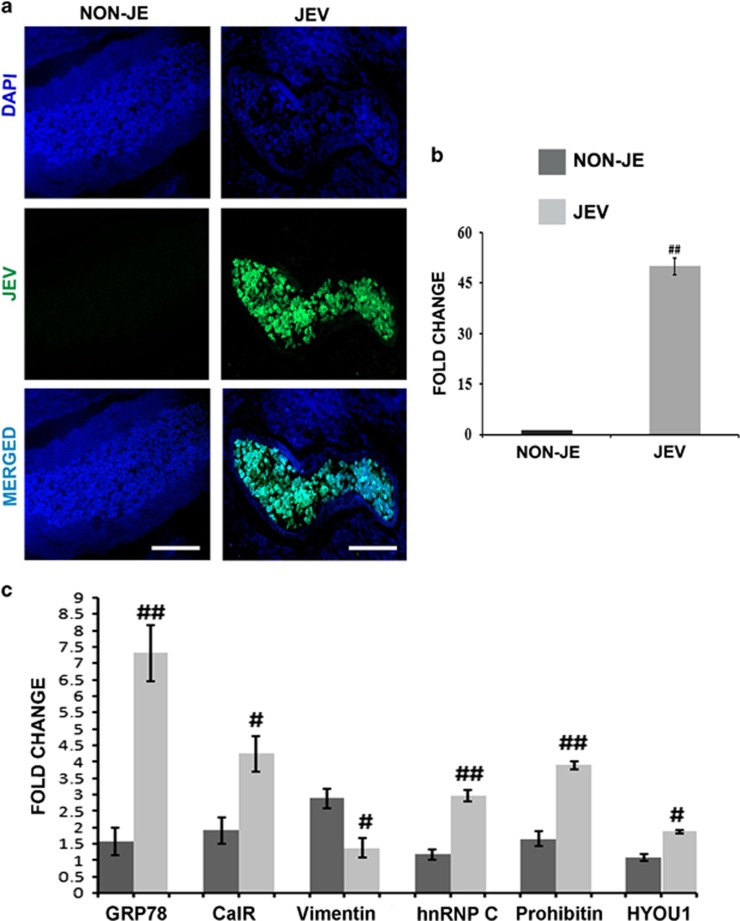
JEV-induced encephalitic and non-encephalitic cases of human autopsy samples (basal ganglia region) showing elevated expression of GRP78, PHB and hnRNPC. (**a**, **b**) Human autopsy samples were first characterized for effective JEV infection through immunohistochemistry (scale bar −50 μm) and real-time PCR. (**c**) Expression profile of proteins detected in mass spectrometry data was re-evaluated by real-time PCR experiment and results show significant upregulation of GRP78, PHB and hnRNPC. Other proteins like Calreticulin and HYOU1 display significant expression. Although in case of Vimentin the data show notable reduction that may be due to the more complex scenario of human brain post JEV infection (^#^*P*<0.01, ^##^*P*<0.001). Data are representative of three independent experiments (mean±S.D.)

Detection of JEV infection in the post mortem autopsy cases of JEV-infected and non-JE samples were administered by immunohistochemistry and qPCR experiments (Figures 7a and b). To get an analogy of host response between *in vitro* cell system and complex human system after JEV infection, we validated the proteomic data in autopsy samples through qPCR. Significant elevation in the GRP78, PHB, hnRNPC mRNA levels along with the mRNA levels of other identified proteins were observed in basal ganglia regions of JE patients when compared to age-matched control of non-JE cases (Figure 7c). Since neurogenic region tissue (sub-ventricular zone) was unavailable in Human Brain Bank of NIMHANS, Bangalore, which is the only brain repository in India, we performed our experiments with basal ganglia tissue only. This is an unavoidable limitation of our current study.

## Discussion

Human neural stem/progenitor cells are a good model to study virus-induced neurodevelopmental disorders. JEV infection in NSPCs creates an alarming situation because of cell cycle arrest and impaired neurogenesis^3^ followed by delayed brain repair, which is responsible for poor morbidity due to neurocognitive anarchy in the JE survivors. Several other reports also indicate the permissiveness of viruses inside NSPCs in both rodent models and human cells. For example, Coxsackievirus B3, an enterovirus, proliferates inside type A progenitors of SVZ region and spreads to the olfactory bulb.^16, 17^ HIV also multiplies well inside NSPCs,^18^ generating HIV associated neurological disorders. Besides these, there are no other reports available showing virus-associated proteomic changes in NSPCs. JEV, like all other flaviviruses, hijack the cellular protein synthesis machinery for its own replication. Therefore it is of immense importance to study the NSPC proteomic profile to generate a glimpse of the host cell proteins post JE infection. In this study, our group reports JEV-induced proteomic alterations and the follow-up consequences in hNS1 cells, which is being used as a novel *in vitro* model of human neural stem/progenitor cell line.

We identified 13 differentially expressed host proteins in hNS1 cells following JEV infection (Table 1). Previous established reports led us to emphasize on ER stress associated UPR and viral element recognition pathways. It is a well-established fact that effectual functioning of ER is indispensable for a cell to survive. ER is pivotal for protein synthesis, folding and assembly. Viral interference reduces the protein folding capacity of ER, thus eliciting UPR. If the cell cannot restore ER homeostasis, UPR results in prolonged stress, which ultimately culminates in cell death. ER dysfunction is the cause of various diseases including ischemia, neurodegeneration and diabetes.^19^ UPR induction is a common phenomenon observed in multiple flaviviral infections like West Nile virus,^20^ hepatitis C virus,^21^ dengue virus,^22^ including JEV. In case of JEV infection, a large amount of viral protein synthesis takes place in ER lumen causing expansion of ER membrane.^11^ GRP78 senses UPR and simultaneously PKR like ER kinase (PERK), Inositol-responsive enzyme 1 (IRE1) and activating transcription factor (ATF6) pathways get activated. JEV has been reported to activate both IRE-1 and PERK branch of UPR.^23, 24^ Since we observed significant presence of GRP78 in proteomic data, we aimed to look upon the status of these UPR initiator proteins in both hNS1 cells and JEV-infected mouse SVZ and found all of them to be upregulated (Figure 2). On the other hand, Sec61 is a multiprotein complex, which is associated with Sec62/Sec63 and GRP78 for protein transport to and from ER.^25^ Accumulated unfolded proteins inside the ER are fated to move to cytosol via retrograde transport for proteosomal degradation via Sec61. We observed increased expression of Sec61/62/63, which is believed to promote retrograde transport of misfolded polypeptides from ER to cytosol for degradation. Hence the induction of ER stress in NSPCs following JEV infection was further confirmed.

There are two plausible consequences of ER stress; either the cell turns towards the survival pathway and restores normal functioning or pro-apoptotic signals prevail in case of unresolved stress. Human cytomegalovirus has been shown to promote apoptosis in NSPCs by enhancing ER stress and mitochondrial malfunction.^14^ A very recent report also portrays death of hNPCs infected by Zika virus at 72 h post infection by activation of caspase 3.^12^ As we observed JEV-induced cytopathic effect in both hNS1 and hNPCs (Figures 1a and 6a), we investigated the status of the apoptotic mediator proteins in these cells. As anticipated, we observed significant increase of caspase proteins in NSPCs post JEV challenge (Figures 3c and 6c). TUNEL-positive hNS1 cells at 72 h post infection (Figure 3a) and elevated ROS (Figure 3b) further strengthen our hypothesis of ER stress-mediated NSPC death. Similar conclusions were obtained in JEV-infected SVZ regions of both young and adult BALB/c mouse (Figure 3c and supplementary Figure S4).

Prohibitin (PHB), an abundant protein identified in this study in hNS1 cells, has previously been reported as a receptor protein mediating Chikungunya virus internalization inside microglia.^26^ It is also reported to interact to C-terminal domain of HIV-1 glycoprotein^27^ and responsible for HCV pathogenesis.^28^ hnRNPC also is reported to interact to poliovirus RNA termini^29^ and takes part in dengue virus replication.^30^ We therefore wanted to explore their role in JEV infection since these proteins were significantly upregulated in our proteomic data. We conducted RNA Co-IP experiment and found out that not only PHB and hnRNPC interact to JEV RNA, but surprisingly GRP78 was also found to be interacting with JEV. To the best of our knowledge, this is the first report showing such RNA–protein interaction (Figure 4a). GRP78 is reported to induce apoptosis by unsequestering PERK, ATF6 and IRE1 after accumulation of misfolded proteins in ER,^31^while PHB is reported to induce apoptosis by means of caspase 3 and caspase 9 over expression in gastric cancer cells.^32^ Therefore, to analyse the role of these three proteins in viral recognition and regulation of apoptosis, we ablated them by siRNA application in hNS1 cells and it revealed decreased virion release (Figure 5c and Supplementary Figure S7) and decreased caspase 3 profile (Figure 5a) post knockdown.

There are many studies that have reported that apart from inducing stress response GRP78 also plays a role in viral internalization. It has been shown to interact with MHC-I molecules and facilitates Coxsackievirus A9 entry in GMK cell lines.^33^ HSP70 and HSP90 along with GRP78 have been shown to be secreted after JEV infection in BHK-21 cells^34^ and are proposed to facilitate mature JEV virion entry through lipid raft by forming a chaperone complex. We also monitored the release of GRP78 along with HSP60, HSP70 and HSP90 in hNS1 secretion post JEV infection (Figures 4c and d). This is supposed to be the first report identifying the release of chaperone proteins post JEV infection by neural stem/progenitor cells.

Evidences point out the release of proinflammatory cytokines like MCP-1, IL-6 during ER stress conditions.^35, 36^ A previous report from our research group has shown proinflammatory cytokine induction in JEV-infected neurospheres.^4^ We also observed elevated TNF-*α*, MCP-1 and IL-6 in mouse sub-ventricular zone and elevated IL-6 in hNS1 cell supernatant (Supplementary Figure S8), which are thought to promote GRP78 and ATF4 expression post JEV infection (Figure 2). IL-6 is also reported to activate the promoter region of PHB gene thereby inducing its transcription in intestinal epithelial cells,^37^ which may be a possible molecular event in JEV infection.

Taken together, from our data we could infer that JEV induces the expression of GRP78, PHB and hnRNPC to promote NSPC death by caspase activation. These expression patterns were validated in the hNPCs isolated from human foetus and significant caspase expression as well (Figure 6). JEV was found to co-localize with each of them inside hNPCs post 72 h of infection (Supplementary Figure S9). Identical results were recorded from encephalitic human patient samples confirming the pivotal role of these proteins in JEV neuropathogenesis (Figure 7).

Last, we wanted to screen GRP78, PHB and hnRNPC expression in other neurotropic RNA viruses namely CHPV, which belongs to Rhabdoviridae family to compare whether neurotropic viruses hijack same mediator proteins to propagate inside NSPCs. To our surprise, we observed significant upregulation of these proteins in hNS1 cells post CHPV infection (Supplementary Figure S6). Through this survey, we are reporting a novel insight into the upheaval of GRP78, PHB and hnRNPC in NSPCs post Rhabdovirus infection.

To summarize, our current study establishes the role of GRP78, PHB and hnRNPC in flaviviral multiplication inside NSPCs. The study also suggests a role of these three proteins in recognition of JEV RNA followed by induction of apoptosis. We also obtained the upregulation of these proteins in CHPV infection. These findings may pave a path for the discovery of therapeutic intervention to protect NSPCs from neurotropic virus attack.

## Material and methods

### Ethics statement

All animal experiments performed were endorsed by the Animal Ethics Committee of National Brain Research Centre (Approval no – NBRC/IAEC/2014/96). Animals were monitored with good care according to the outline of Committee for the Purpose of Control and Supervision of Experiments on Animals (CPCSEA), Ministry of Environment and Forestry, Government of India.

### Virus propagation

GP78 strain of JE virus was propagated on suckling BALB/c mice and their brains were harvested after manifestation of clinical symptoms of encephalitis. Virus isolation and titration were done as reported earlier.^4^

### Cell culture

#### hNS1 culture

hNS1 cell line is a kind gift from Dr Alberto Martínez-Serrano, Centre of Molecular Biology Severo Ochoa, Autonomous University of Madrid, Spain. hNS1 (formerly called HNSC.100, a model cell line of hNSCs) is a human embryonic forebrain-derived, multipotent, clonal cell line of neural stem cells with stable cell cycle length, mitotic potential and differentiation capacity.^38, 39^ hNS1 cells were grown in poly-D-lysine-(Sigma, USA) coated flasks in a chemically defined DMEM-F12 medium (Invitrogen, CA, USA) supplemented with 20 ng/ml each of rhEGF (epidermal growth factor) and rhFGF (fibroblast growth factor) (R&D Systems, USA), BSA (Sigma), Gentamicin and N2 supplement (Invitrogen). Upon reaching confluency, cells were infected with JEV at an MOI of 5. Cells were harvested at the intervals of 24, 48 and 72 h and analysed for viral antigen of JEV by means of real-time PCR and plaque assay. After 72 h post infection, significant changes in both real-time PCR and plaque assay were observed along with visible changes in cell morphology. This led us to choose 72 h post infection of cell lines as the optimal time point for further studies.

#### Human neural precursor cells (hNPCs) culture

Human neural precursor cells (hNPCs) were cultured according to the earlier published protocol.^40^ Isolation of hNPCs from aborted human foetus was carried out following ethical guidelines of NBRC, Department of Biotechnology (DBT) and Indian Council of Medical Research (ICMR) for Stem Cell Research. Briefly cells were cultured in poly-D-lysine (Sigma) coated flasks in neurobasal media (Invitrogen) supplemented with Neural survival factor (Lonza, USA), N2 supplement (Invitrogen) 25 ng/ml bovine fibroblast growth factor (bFGF) (Sigma-Aldrich, USA) and 20 ng/ml EGF (Sigma-Aldrich).These cells were also infected with JEV at MOI 5 and cell samples were harvested for protein or RNA isolation or for immunocytochemistry at 72 h post infection.

#### Protein extraction, two-dimensional gel electrophoresis and mass spectrometry

hNS1 cells were grown at a density of 10^7^ cells. Upon confluency, cells were kept in growth factor-free media followed by JEV infection. After 72 h cells were harvested, pelleted and sonicated (Biologs, Inc, USA) in RIPA buffer for protein isolation. Protein concentration was determined using bicinchoninic acid (Sigma) protein estimation method. Two-dimensional gel electrophoresis was performed as described earlier.^8, 41^ Briefly, 200 μg of protein was precipitated overnight by using 10% TCA followed by two acetone washes at 10000 g for 10 min at 4 °C. Protein pellet thus obtained was suspended in 150 μl of sample rehydration buffer containing 7 M urea, 4% CHAPS and 50 mM DTT (Bio-Rad). IPG strips of 7 cm size with a pH range from 3 to 10 were used for all the experiments. The strips were rehydrated overnight with 200 μg of protein in 150 μl of rehydration buffer. IPG strips were then focussed on a Protean i12^TM^ IEF cell (Bio-Rad) for 10000 VHr at 20 °C (first dimension). After focussing, strips were incubated with equilibration buffers I and II (Bio-Rad) for 10 min each, followed by 12% SDS-PAGE run (second dimension).

Protein spots were visualized by staining with Coomassie blue R-250 (Sigma). Gel images were captured by LI-COR odyssey imager (LI-COR Biosciences, USA). Images of three biological replicates (control and JEV infected) were used for automatic spot detection by PD Quest 2D Analysis Software (Hercules, CA, USA). Spot intensities were normalized by mean values from biological replicates (*t*-test analysis was done by GraphPad Prism Software) and protein spots showing altered expression between control and infected groups (ratio >=1.5) were marked, excised and sent for mass spectrometry. The mass spectrometry analysis was done by Sandor Life Sciences Pvt. Ltd, Hyderabad, India.

#### JE infection in animals

Ten-day-old BALB/c mice pups of either sex were divided randomly into two groups. One group of mice were intraperitoneally infected with 3 × 10^5^ pfu of JEV. The other group marked as control received equal volume of sterile PBS. Post infection JEV-infected animals started developing neurological symptoms post day 5 of infection. At day 7 post infection, animal SVZ samples were collected along with age-matched controls. Animals of both groups were sacrificed for the isolation of subventricular zone in ice-cold sterile PBS followed by protein or RNA isolation. For SVZ isolation, briefly, animals were first euthanized with ketamine and perfused with ice-cold PBS. Head was removed by giving a cut above the cervical spinal cord region. Then skull was removed and coronal slices (~ 500 μm) of the brain were prepared. Thereafter, slices were kept in a Petri dish in chilled PBS. The subventricular zone was dissected out from the selective slices under the dissection microscope.

#### RNA isolation and qPCR

RNA from SVZ region was extracted by Trizol (Sigma) reagent followed by chloroform and isopropanol treatment. RNA from hNS1 cells, foetal progenitor cells as well as SVZ tissue, was extracted by RNAeasy Mini Kit (Qiagen, Hamburg, Germany). cDNA was synthesized from the isolated RNA using advantage RT-PCR Kit (Clontech, Mountain View, USA). The conditions for qPCR reactions were 95 °C for 5 min (1 cycle), 95 °C for 30 s, annealing temperature for 30 s and 72 °C for 30 s (45 cycles). The results were normalized with respective human or mouse GAPDH by ΔΔCT method and are depicted as fold change over mock-infected control. Primers used to amplify JEV GP78 were F (5′-3′) TTGACAATCATGGCAAACG; R (5′-3′) CCCAACTTGCGCTGAATAA. Rest of the primer sequences are provided in Supplementary Tables S1 and S2.

#### RNA isolation from human samples

Formalin-fixed paraffin embedded sections from the autopsied human brain cases of JE (basal ganglia region) were obtained from the archives of Human Brain Bank, NIMHANS, Bangalore (CSF positive for JE-IgM), non-JE control samples were from subjects who met with road traffic accidents with minimal trauma to brain. The RNA extraction process from these tissue samples followed the formerly published guidelines.^42^ cDNA preparation and qPCR protocols were identical for the tissues, similar to cell lines.

#### Immunoblot

Total protein was isolated from SVZ tissue, hNS1 cells and foetal progenitor cells (control and JEV infected) and estimated by bicinchoninic acid method. 20 μg of each of the sample was separated in SDS PAGE followed by transfer into nitrocellulose membrane. They were incubated with primary antibodies overnight at 4 °C (dilutions are enlisted in Supplementary Table S3. After PBST wash in the next day, blots were incubated with suitable secondary antibodies (1:5000, Vector Laboratories, USA) followed by washing and developing through ECL kit (Millipore, CA, USA). Images were captured in Chemigenius Bioimaging System (Syngene, Cambridge, UK). To ensure equal loading, blots were stripped and reincubated with beta actin (1:10000, Sigma).

#### Protein isolation from cell culture supernatant

hNS1 cells were infected with JEV and cell culture supernatants post 24, 48 and72 h of infection were collected along with mock-infected media. Trichloroacetic acid (Sigma, USA) was added to respective media samples at a ratio 1:4 (TCA:Media=1:4) and kept O/N at 4 °C. Precipitated proteins were collected by centrifugation followed by Acetone (Merck, USA) wash. After air drying, protein pellets were resuspended in Urea-CHAPS buffer (8 M urea, 2% CHAPS) and protein concentration was determined by Bradford Assay.

### ROS assay

The elevation of ROS production in hNS1 cells of both uninfected and infected groups was measured by 5(and 6)-chlromethyl-20, 70-dichlorodihydrofluoresceindiacetate (DCFDA; Sigma). Inside the cell, this compound is broken down to yield a fluorescent intermediate named dichlorofluorescein. Uninfected and infected cells were treated with 20 μM DCFDA and incubated in dark for 30 min. The cells were then pelleted down and resuspended in 1X PBS. The intracellular fluorescence was measured through flow cytometry (BD Biosciences, San Diego, CA, USA). Similarly, another set of cells were again treated with N-acetyl L-cysteine (scavenger of ROS; Sigma, USA) prior to JEV infection and ROS production was measured.

### RNA Co-IP and sequencing

Interaction of JEV RNA with GRP78, PHB and hnRNPC was analysed by the method described earlier.^42^ Briefly, non-infected or infected hNS1 cells were treated with 1% formaldehyde for 10 min at RT in Hula mixer (Invitrogen) followed by addition of 0.25 M glycine of pH 7.0. These cross-linked cells were sonicated to get the protein complex. Precleared lysate was obtained by addition of dynabeads containing protein G (Novex, Life Technologies, USA). The beads were coated with respective antibodies and allowed to rotate alongwith precleared supernatant at RT for 90 min. The beads were washed thoroughly with antibody binding and washing buffer supplied in the immunoprecipitation kit. The complexes were eluted by the elution buffer. RNA was extracted from the elute samples by conventional method using Trizol reagent (Sigma). RNAs obtained were used in RT-PCR with JEV primers and reaction without reverse transcriptase was used as control. The PCR products were analysed in 2% agarose gel. Purified PCR products (PCR Purification Kit, Qiagen) of JE-positive samples were sent for sequencing. Sequencing was done by Invitrogen Bioservices India Pvt. Ltd.

### Intracellular staining of JEV

hNS1 cells were harvested every 24 h of JEV treatment till 72 h. Control and infected cells were treated with Cytofix solution and Permeabilisation buffer (BD Biosciences) sequentially. Cells were then washed twice and resuspended in wash buffer (PBS with 1% BSA). Primary antibody was added to each experimental cell suspension (JEV Nakayama strain; Chemicon, USA; 1∶100 dilutions) and incubated at room temperature for 1 h. After washing, cells were incubated with FITC conjugated secondary antibody (Invitrogen) for 30 min at room temperature. After final wash, cells were resuspended in 500 μl of FACS buffer and analysed in Cell Quest Pro software in FACS Calibur (BD Biosciences).

### Immunofluorescent staining

hNS1 and foetal progenitor cells were seeded in four-well poly-D-lysine coated chamber slides and infected with JEV as described previously. After 72 h of infection, cells were fixed with 4% paraformaldehyde for 20 min followed by PBS washing and blocking with 5% horse or goat serum (Vector Laboratories) according to the antibodies used. Cells were incubated with primary antibodies (dilution used 1:250 for all) overnight at 4 °C in humid chamber. Next day cells were washed in PBST and incubated with suitable fluorochrome conjugated secondary antibodies (1:500) at room temperature for 1 h (antibody dilutions are mentioned in Supplementary Table S3). After final washes, cells were mounted in DAPI (Vector Laboratories) and images were captured with Zeiss Apotome Microscope (Zeiss, Germany).

### siRNA silencing

Endonuclease prepared short interfering RNA (esiRNA) of human GRP78, PHB, hnRNPC and scrambled negative control were obtained from Sigma-Aldrich. 60 pmol of GRP78 and hnRNPC esiRNA were used for transfection of 60% confluent hNS1 using Lipofectamine RNAi Max (Invitrogen) according to manufacturer's instruction. For PHB transfection, 1 μg of esiRNA was used and transfection was carried out using Neuromag Transfection Reagent (OZ Biosciences, San Diego, USA) following supplier's protocol. Post 24 h of transfection, cells were infected with JEV at an MOI of 5 and samples were withdrawn for western blot analysis after 72 h of infection.

Cell culture supernatants of these experiments were used for viral RNA isolation using high pure viral nucleic acid kit (Roche Life Science, USA). Equal quantity of RNA from each of transfected and non-transfected samples were used for cDNA synthesis followed by qPCR of viral RNA. qRT-PCR products indicative of viral replication were analysed through agarose gel.

### Cytokine assay by bead array

IL-12, TNF-*α*, IFN-*γ*, MCP-1, IL-10 and IL-6 concentrations were measured in mouse subventricular zone (both uninfected and infected) by flow cytometry using a mouse inflammation CBA kit (BD Biosciences) as per the manufacturer's instructions. On the other hand, IL-12, TNF-*α*, IL-1*β*, IL-6, IL-8 and IL-10 concentrations were measured in cell supernatants of hNS1 (both uninfected and infected) by flow cytometry using a human inflammatory cytokine bead array kit (BD Biosciences) as per the manufacturer's instructions. Data in both cases were obtained through FACS Calibur (Becton Dickinson, San Diego, CA, USA).

### Statistical analysis

Data are represented as mean±S.D. of three independent experiments in triplicate (*n*=3). Statistical significance was evaluated using Student's *t*-test or one way analysis of variance (ANOVA) followed by Holm–Sidak *post hoc* test. *P* value<0.05 was considered to be statistically significant.

## Figures and Tables

**Table 1:** Proteins showing differential expression after JEV infection, identified by MS/MS analysis of gel excised spots

*Spot No*	*Protein ID*^a^	*Matched peptides*	*% coverage*	*Mowse score*^b^	*MW (theor/obs)*	*pI*
1	Uncharacterized protein C19orf45 (Homo sapiens) NP_940936	K.TTMGSDYCPSEWR.Q K.APNLHLQQSYLPR.G K.DEFPFKYQGPAALR.L	14	72	57979/50000	9.04
2	Lamin isoform D (Homo sapiens) NP_001244303	R.VAVEEVDEEGKFVR.L R.EFESRLADALQELR.A R.IRIDSLSAQLSQLQK.Q	17	84	64253/65000	6.30
3	Prohibitin (Homo sapiens) NP_001268425	R.FDAGELITQR.E K.DLQNVNITLR.I R.ILFRPVASQLPR.I	26	82	29859/25000	5.57
4	Mitochondrial ATP synthase, H+ transporting F1 complex beta subunit (Homo sapiens) NP_001677	R.LVLEVAQHLGESTVR.T R.IMDPNIVGSEHYDVAR.G R.AIAELGIYPAVDPLDSTSR.I	28	94	48083/51000	4.95
5	Calreticulin precursor (Homo sapiens) NP_004334	K.KVHVIFNYK.G K.EQFLDGDGWTSR.W K.IKDPDASKPEDWDER.A	13	72	48283/52000	4.29
6	78 kDa glucose-regulated protein precursor (Homo sapiens). NP_005338	R.IINEPTAAAIAYGLDKR.E K.VTHAVVTVPAYFNDAQR.Q R.IEIESFYEGEDFSETLTR.A	24	153	72402/75000	5.07
7	Vimentin (Homo sapiens) NP_003371	K.FADLSEAANRNNDALR.Q R.ETNLDSLPLVDTHSKR.T R.ISLPLPNFSSLNLR.E	40	160	53676/60000	5.06
8	Heterogeneous nuclear ribonucleoprotein C (C1/C2) (Homo sapiens) NP_001070911	R.MYSYPAR. R.VPPPPPIAR.A K.GFAFVQYVNER.N	27	72	19079/30000	10.22
9	Zinc finger protein 224 (Homo sapiens) NP_037530	K.IWMMKTAIQR.E R.LNLDMHQRVHMGEK.T R.VHSGEKPFKCEECGK.G	16	67	84881/40000	8.96
10	RNA polymerase II subunit A C-terminal domain phosphatase. (Homo sapiens) NP_430255	R.SMEAHNILSK.R K.DLFDLILTCEER. K.TTYDQMYNDLLRK.D	25	56	23016/100000	5.16
11	Actin, cytoplasmic 1 (Homo sapiens) NP_001092	K.IWHHTFYNELR.V K.QEYDESGPSIVHR.K K.SYELPDGQVITIGNER.F	20	74	38950/40000	5.19
12	HYOU1 protein (Homo sapiens) NP_001124463	K.QADNPHVALYQAR.F R.FPEHELTFDPQR.Q R.DAVVYPILVEFTR.E	11	73	73229/100000	5.74
13	Nebulin-related anchoring protein (Homo sapiens) NP_932326	R.ANAANLSEVKYK.E K.KAYGLQSELQYK.A K.GSFPAMITPAYQR.A	6	69	193924/40000	9.22

a NCBI accession number of identified proteins is mentioned.

b MS/MS data of three peptides for each spot was searched against NCBI database in the taxonomy group of *Homo sapiens* using Mascot tool. A total of three peptides were subjected to MS/MS analysis and the fragment ion data were searched against the data base.

## References

[bib1] Thounaojam MC, Kundu K, Kaushik DK, Swaroop S, Mahadevan A, Shankar SK et al. MicroRNA 155 regulates Japanese encephalitis virus-induced inflammatory response by targeting Src homology 2-containing inositol phosphatase 1. J Virol 2014; 88: 4798–4810. 24522920 10.1128/JVI.02979-13 PMC3993824

[bib2] Das S, Chakraborty S, Basu A. Critical role of lipid rafts in virus entry and activation of phosphoinositide 3' kinase/Akt signaling during early stages of Japanese encephalitis virus infection in neural stem/progenitor cells. J Neurochem 2010; 115: 537–549. 20722967 10.1111/j.1471-4159.2010.06951.x

[bib3] Das S, Basu A. Japanese encephalitis virus infects neural progenitor cells and decreases their proliferation. J Neurochem 2008; 106: 1624–1636. 18540995 10.1111/j.1471-4159.2008.05511.x

[bib4] Das S, Ghosh D, Basu A. Japanese encephalitis virus induce immuno-competency in neural stem/progenitor cells. PloS One 2009; 4: e8134. 19956550 10.1371/journal.pone.0008134 PMC2780913

[bib5] Munday DC, Surtees R, Emmott E, Dove BK, Digard P, Barr JN et al. Using SILAC and quantitative proteomics to investigate the interactions between viral and host proteomes. Proteomics 2012; 12: 666–672. 22246955 10.1002/pmic.201100488

[bib6] Issac TH, Tan EL, Chu JJ. Proteomic profiling of chikungunya virus-infected human muscle cells: reveal the role of cytoskeleton network in CHIKV replication. J Proteomics 2014; 108: 445–464. 24933005 10.1016/j.jprot.2014.06.003

[bib7] Pando-Robles V, Oses-Prieto JA, Rodriguez-Gandarilla M, Meneses-Romero E, Burlingame AL, Batista CV. Quantitative proteomic analysis of Huh-7 cells infected with Dengue virus by label-free LC-MS. J Proteomics 2014; 111: 16–29. 25009145 10.1016/j.jprot.2014.06.029

[bib8] Sengupta N, Ghosh S, Vasaikar SV, Gomes J, Basu A. Modulation of neuronal proteome profile in response to Japanese encephalitis virus infection. PloS One 2014; 9: e90211. 24599148 10.1371/journal.pone.0090211 PMC3943924

[bib9] Kim I, Xu W, Reed JC. Cell death and endoplasmic reticulum stress: disease relevance and therapeutic opportunities. Nat Rev Drug Discov 2008; 7: 1013–1030. 19043451 10.1038/nrd2755

[bib10] Lu TH, Su CC, Chen YW, Yang CY, Wu CC, Hung DZ et al. Arsenic induces pancreatic beta-cell apoptosis via the oxidative stress-regulated mitochondria-dependent and endoplasmic reticulum stress-triggered signaling pathways. Toxicol Lett 2011; 201: 15–26. 21145380 10.1016/j.toxlet.2010.11.019

[bib11] Su HL, Liao CL, Lin YL. Japanese encephalitis virus infection initiates endoplasmic reticulum stress and an unfolded protein response. J Virol 2002; 76: 4162–4171. 11932381 10.1128/JVI.76.9.4162-4171.2002 PMC155064

[bib12] Tang H, Hammack C, Ogden SC, Wen Z, Qian X, Li Y et al. Zika virus infects human cortical neural progenitors and attenuates their growth. Cell Stem Cell 2016; 18: 587–590. 26952870 10.1016/j.stem.2016.02.016 PMC5299540

[bib13] Xu J, Nash RJ, Frey TK. Cellular responses to Sindbis virus infection of neural progenitors derived from human embryonic stem cells. BMC Res Notes 2014; 7: 757. 25343994 10.1186/1756-0500-7-757 PMC4307679

[bib14] Nakamura H, Liao H, Minami K, Toyoda M, Akutsu H, Miyagawa Y et al. Human cytomegalovirus induces apoptosis in neural stem/progenitor cells derived from induced pluripotent stem cells by generating mitochondrial dysfunction and endoplasmic reticulum stress. Herpesviridae 2013; 4: 2. 24144363 10.1186/2042-4280-4-2 PMC3875896

[bib15] Miner JJ, Diamond MS. Understanding how Zika virus enters and infects neural target cells. Cell Stem Cell 2016; 18: 559–560. 27152436 10.1016/j.stem.2016.04.009

[bib16] Feuer R, Mena I, Pagarigan RR, Harkins S, Hassett DE, Whitton JL. Coxsackievirus B3 and the neonatal CNS: the roles of stem cells, developing neurons, and apoptosis in infection, viral dissemination, and disease. Am J Pathol 2003; 163: 1379–1393. 14507646 10.1016/S0002-9440(10)63496-7 PMC1868316

[bib17] Feuer R, Pagarigan RR, Harkins S, Liu F, Hunziker IP, Whitton JL. Coxsackievirus targets proliferating neuronal progenitor cells in the neonatal CNS. J Neuroscience: The Official Journal of the Society for Neuroscience 2005; 25: 2434–2444. 10.1523/JNEUROSCI.4517-04.2005 PMC6726081 15745971

[bib18] Lawrence DM, Durham LC, Schwartz L, Seth P, Maric D, Major EO. Human immunodeficiency virus type 1 infection of human brain-derived progenitor cells. J Virol 2004; 78: 7319–7328. 15220405 10.1128/JVI.78.14.7319-7328.2004 PMC434111

[bib19] Kaufman RJ. Orchestrating the unfolded protein response in health and disease. J Clin Invest 2002; 110: 1389–1398. 12438434 10.1172/JCI16886 PMC151822

[bib20] Medigeshi GR, Lancaster AM, Hirsch AJ, Briese T, Lipkin WI, Defilippis V et al. West Nile virus infection activates the unfolded protein response, leading to CHOP induction and apoptosis. J Virol 2007; 81: 10849–10860. 17686866 10.1128/JVI.01151-07 PMC2045561

[bib21] Chan SW. Unfolded protein response in hepatitis C virus infection. Front Microbiol 2014; 5: 233. 24904547 10.3389/fmicb.2014.00233 PMC4033015

[bib22] Umareddy I, Pluquet O, Wang QY, Vasudevan SG, Chevet E, Gu F. Dengue virus serotype infection specifies the activation of the unfolded protein response. Virol J 2007; 4: 91. 17888185 10.1186/1743-422X-4-91 PMC2045667

[bib23] Bhattacharyya S, Vrati S. The Malat1 long non-coding RNA is upregulated by signalling through the PERK axis of unfolded protein response during flavivirus infection. Sci Rep 2015; 5: 17794. 26634309 10.1038/srep17794 PMC4669524

[bib24] Bhattacharyya S, Sen U, Vrati S. Regulated IRE1-dependent decay pathway is activated during Japanese encephalitis virus-induced unfolded protein response and benefits viral replication. J Gen Virol 2014; 95(Pt 1): 71–79. 24114795 10.1099/vir.0.057265-0

[bib25] Romisch K. Surfing the Sec61 channel: bidirectional protein translocation across the ER membrane. J Cell Sci 1999; 112 Pt 23 4185–4191. 10564637 10.1242/jcs.112.23.4185

[bib26] Wintachai P, Wikan N, Kuadkitkan A, Jaimipuk T, Ubol S, Pulmanausahakul R et al. Identification of prohibitin as a Chikungunya virus receptor protein. J Med Virol 2012; 84: 1757–1770. 22997079 10.1002/jmv.23403

[bib27] Emerson V, Holtkotte D, Pfeiffer T, Wang IH, Schnolzer M, Kempf T et al. Identification of the cellular prohibitin 1/prohibitin 2 heterodimer as an interaction partner of the C-terminal cytoplasmic domain of the HIV-1 glycoprotein. J Virol 2010; 84: 1355–1365. 19906925 10.1128/JVI.01641-09 PMC2812343

[bib28] Dang SS, Sun MZ, Yang E, Xun M, Ma L, Jia ZS et al. Prohibitin is overexpressed in Huh-7-HCV and Huh-7.5-HCV cells harboring *in vitro* transcribed full-length hepatitis C virus RNA. Virol J 2011; 8: 424. 21896168 10.1186/1743-422X-8-424 PMC3180425

[bib29] Ertel KJ, Brunner JE, Semler BL. Mechanistic consequences of hnRNP C binding to both RNA termini of poliovirus negative-strand RNA intermediates. J Virol 2010; 84: 4229–4242. 20164237 10.1128/JVI.02198-09 PMC2863767

[bib30] Dechtawewat T, Songprakhon P, Limjindaporn T, Puttikhunt C, Kasinrerk W, Saitornuang S et al. Role of human heterogeneous nuclear ribonucleoprotein C1/C2 in dengue virus replication. Virol J 2015; 12: 14. 25890165 10.1186/s12985-014-0219-7 PMC4351676

[bib31] Szegezdi E, Logue SE, Gorman AM, Samali A. Mediators of endoplasmic reticulum stress-induced apoptosis. EMBO Rep 2006; 7: 880–885. 16953201 10.1038/sj.embor.7400779 PMC1559676

[bib32] Zhang L, Ji Q, Ni ZH, Sun J. Prohibitin induces apoptosis in BGC823 gastric cancer cells through the mitochondrial pathway. Asian Pac J Cancer Prev: APJCP 2012; 13: 3803–3807. 23098474 10.7314/apjcp.2012.13.8.3803

[bib33] Triantafilou K, Fradelizi D, Wilson K, Triantafilou M. GRP78, a coreceptor for coxsackievirus A9, interacts with major histocompatibility complex class I molecules which mediate virus internalization. J Virol 2002; 76: 633–643. 11752154 10.1128/JVI.76.2.633-643.2002 PMC136810

[bib34] Wu YP, Chang CM, Hung CY, Tsai MC, Schuyler SC, Wang RY. Japanese encephalitis virus co-opts the ER-stress response protein GRP78 for viral infectivity. Virol J 2011; 8: 128. 21418596 10.1186/1743-422X-8-128 PMC3071342

[bib35] Kolattukudy PE, Niu J. Inflammation, endoplasmic reticulum stress, autophagy, and the monocyte chemoattractant protein-1/CCR2 pathway. Circ Res 2012; 110: 174–189. 22223213 10.1161/CIRCRESAHA.111.243212 PMC3265021

[bib36] O'Neill CM, Lu C, Corbin KL, Sharma PR, Dula SB, Carter JD et al. Circulating levels of IL-1B+IL-6 cause ER stress and dysfunction in islets from prediabetic male mice. Endocrinology 2013; 154: 3077–3088. 23836031 10.1210/en.2012-2138 PMC3749476

[bib37] Theiss AL, Obertone TS, Merlin D, Sitaraman SV. Interleukin-6 transcriptionally regulates prohibitin expression in intestinal epithelial cells. J Biol Chem 2007; 282: 12804–12812. 17324931 10.1074/jbc.M609031200

[bib38] Villa A, Navarro-Galve B, Bueno C, Franco S, Blasco MA, Martinez-Serrano A. Long-term molecular and cellular stability of human neural stem cell lines. Exp Cell Res 2004; 294: 559–570. 15023542 10.1016/j.yexcr.2003.11.025

[bib39] Villa A, Navarro B, Martinez-Serrano A. Genetic perpetuation of *in vitro* expanded human neural stem cells: cellular properties and therapeutic potential. Brain Res Bull 2002; 57: 789–794. 12031275 10.1016/s0361-9230(01)00776-6

[bib40] Fatima M, Kumari R, Schwamborn JC, Mahadevan A, Shankar SK, Raja R et al. Tripartite containing motif 32 modulates proliferation of human neural precursor cells in HIV-1 neurodegeneration. Cell Death Differ 2016; 23: 776–786. 26586575 10.1038/cdd.2015.138 PMC4832097

[bib41] Ghosh S, Mukherjee S, Sengupta N, Roy A, Dey D, Chakraborty S et al. Network analysis reveals common host protein/s modulating pathogenesis of neurotropic viruses. Sci Rep 2016; 6: 32593. 27581498 10.1038/srep32593 PMC5007645

[bib42] Nazmi A, Mukherjee S, Kundu K, Dutta K, Mahadevan A, Shankar SK et al. TLR7 is a key regulator of innate immunity against Japanese encephalitis virus infection. Neurobiol Dis 2014; 69: 235–247. 24909816 10.1016/j.nbd.2014.05.036

