# Supplementary figures and images for: Japanese encephalitis virus induces human neural stem/progenitor cell death by elevating GRP78, PHB and hnRNPC through ER stress
Source: Cell Death Dis. 2017 Jan 19;8(1):e2556–. doi: 10.1038/cddis.2016.394 (PMC5386351; doi:10.1038/cddis.2016.394)

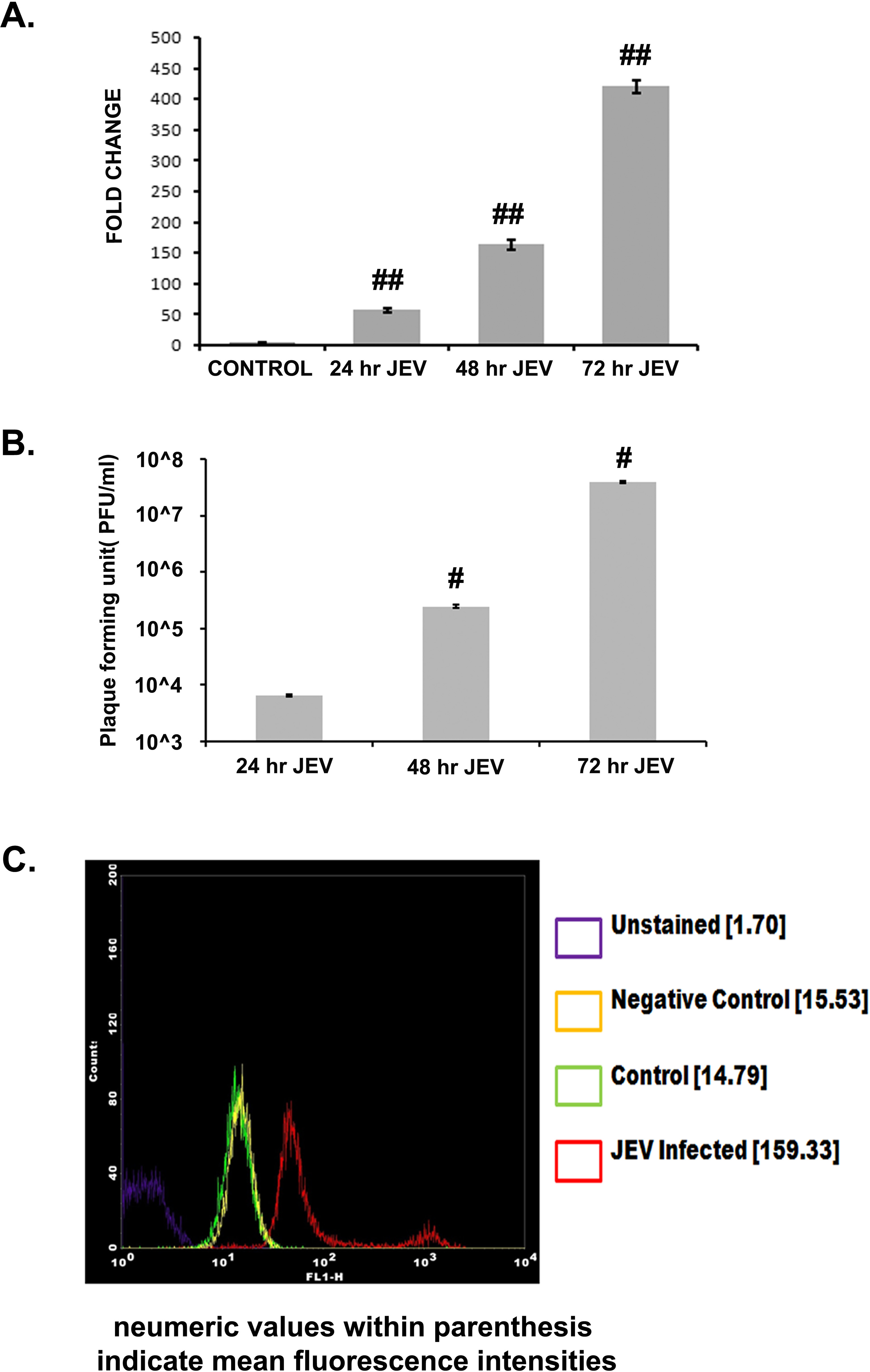

Supplement: Supplementary Figure S1 [file cddis2016394x1.tif]

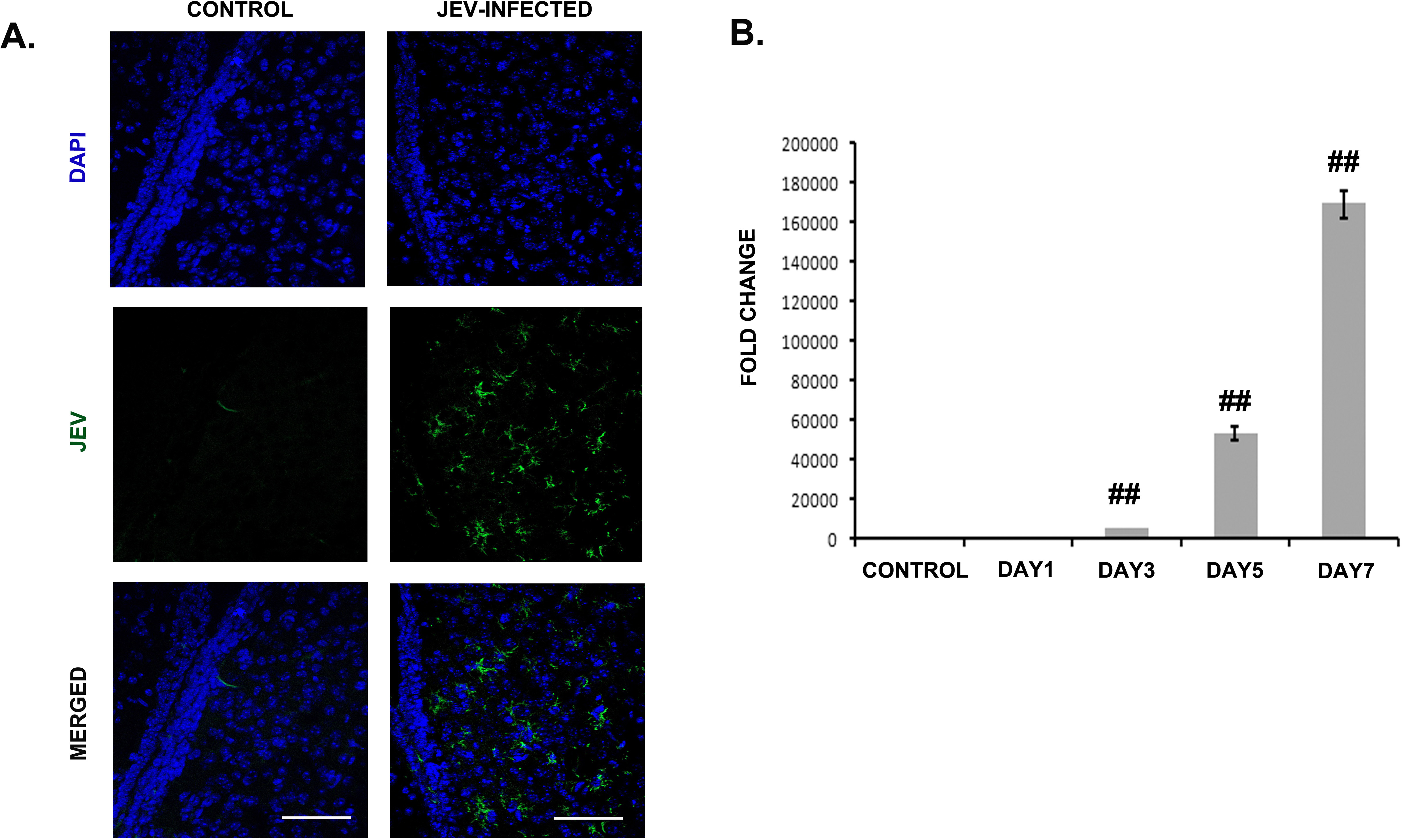

Supplement: Supplementary Figure S2 [file cddis2016394x2.tif]

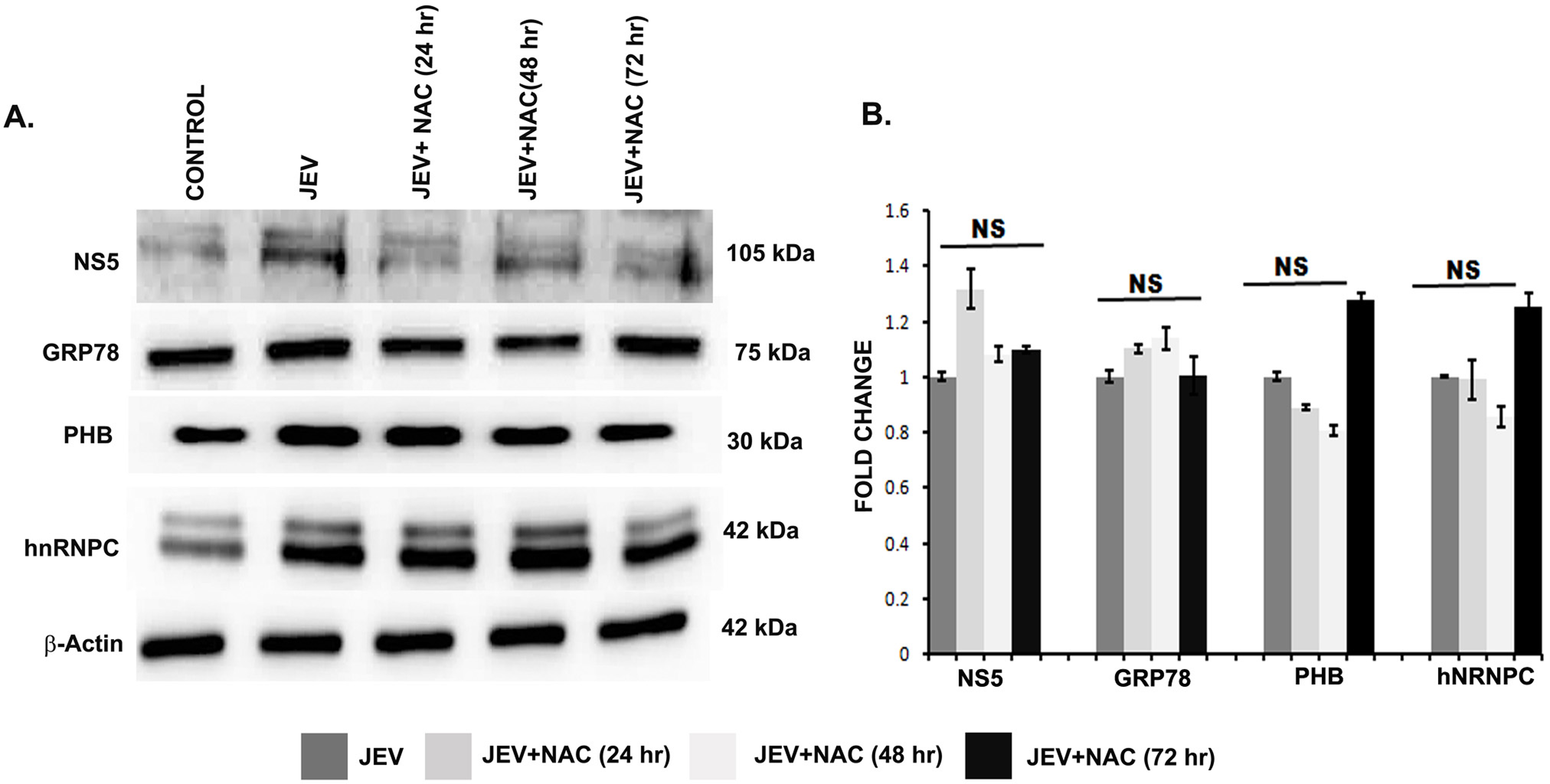

Supplement: Supplementary Figure S3 [file cddis2016394x3.tif]

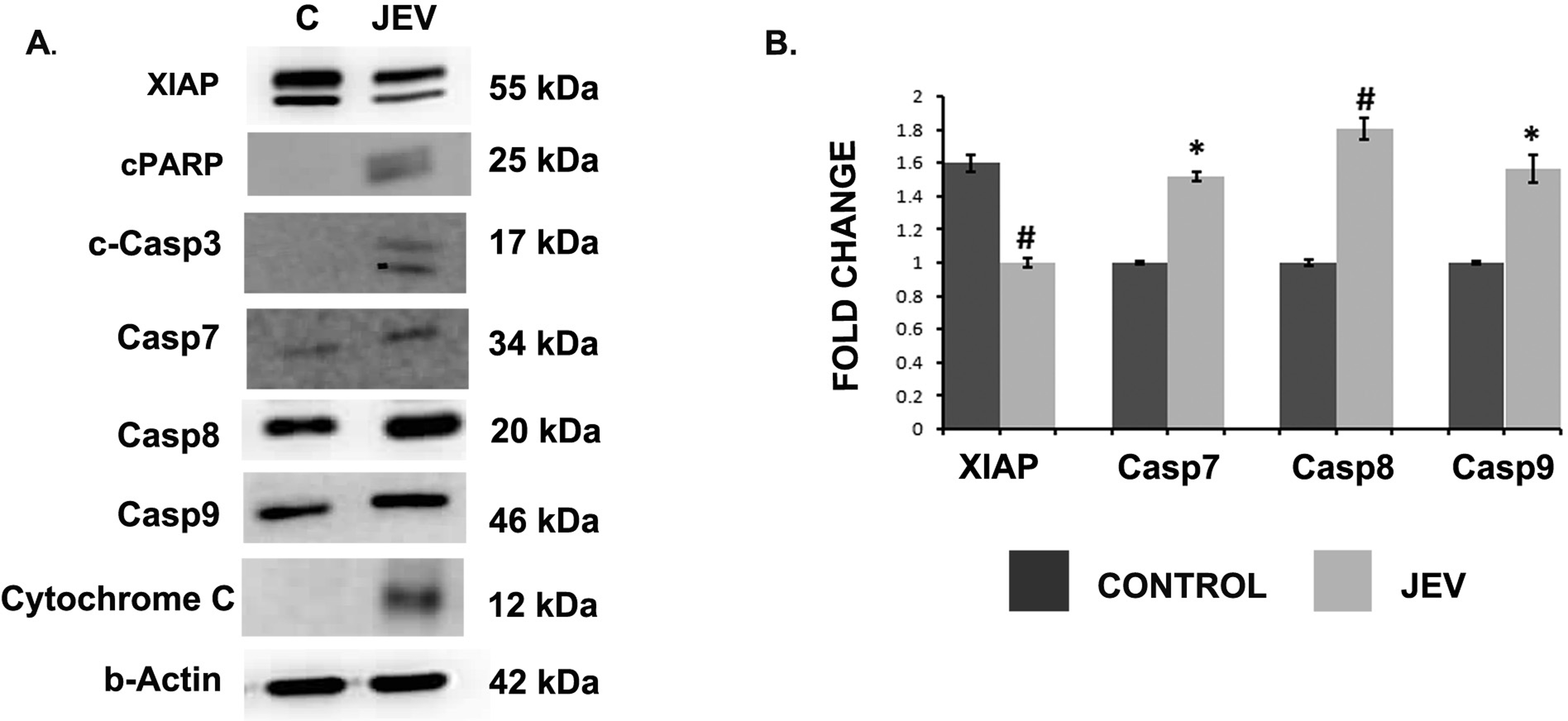

Supplement: Supplementary Figure S4 [file cddis2016394x4.tif]

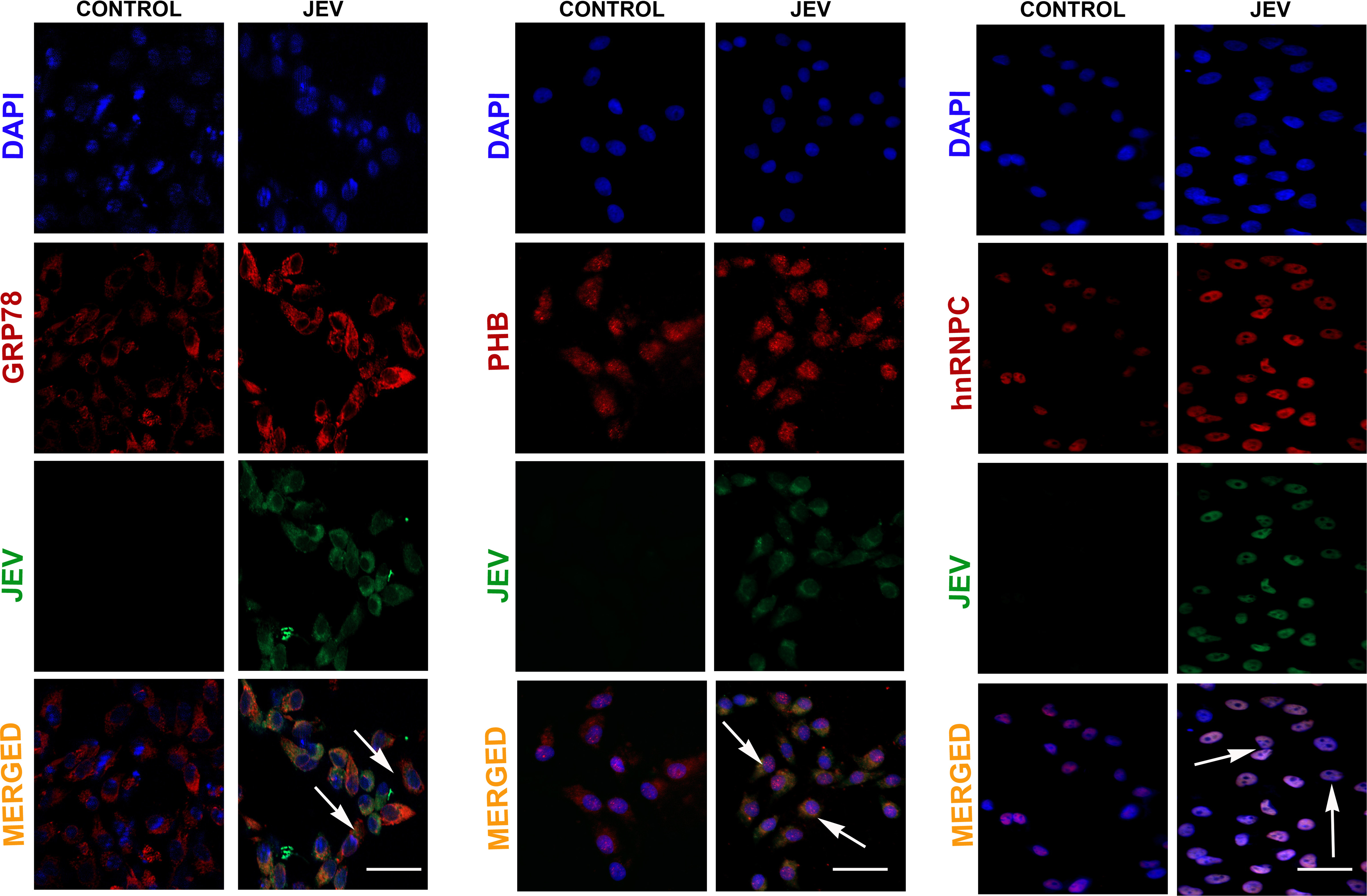

Supplement: Supplementary Figure S5 [file cddis2016394x5.tif]

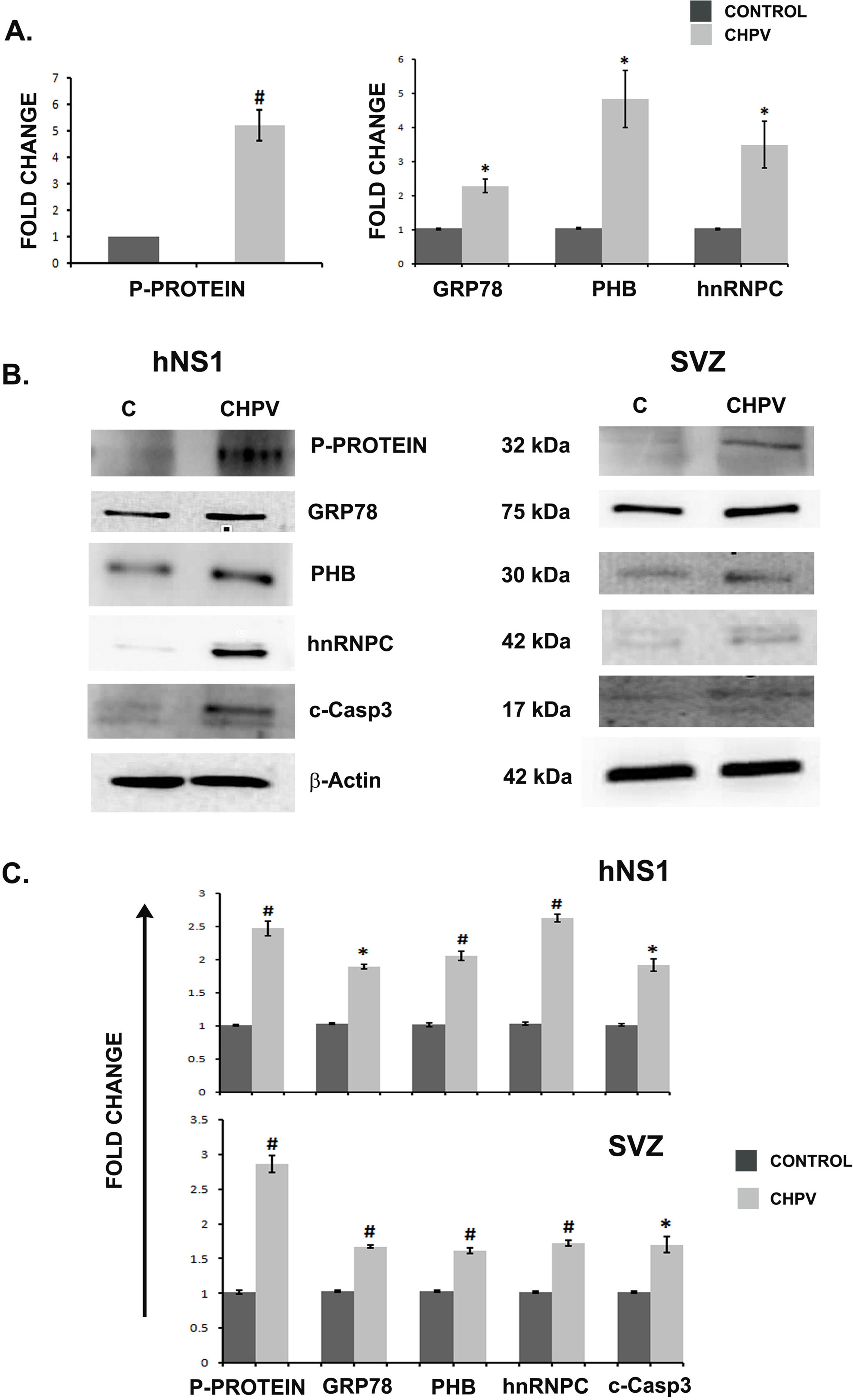

Supplement: Supplementary Figure S6 [file cddis2016394x6.tif]

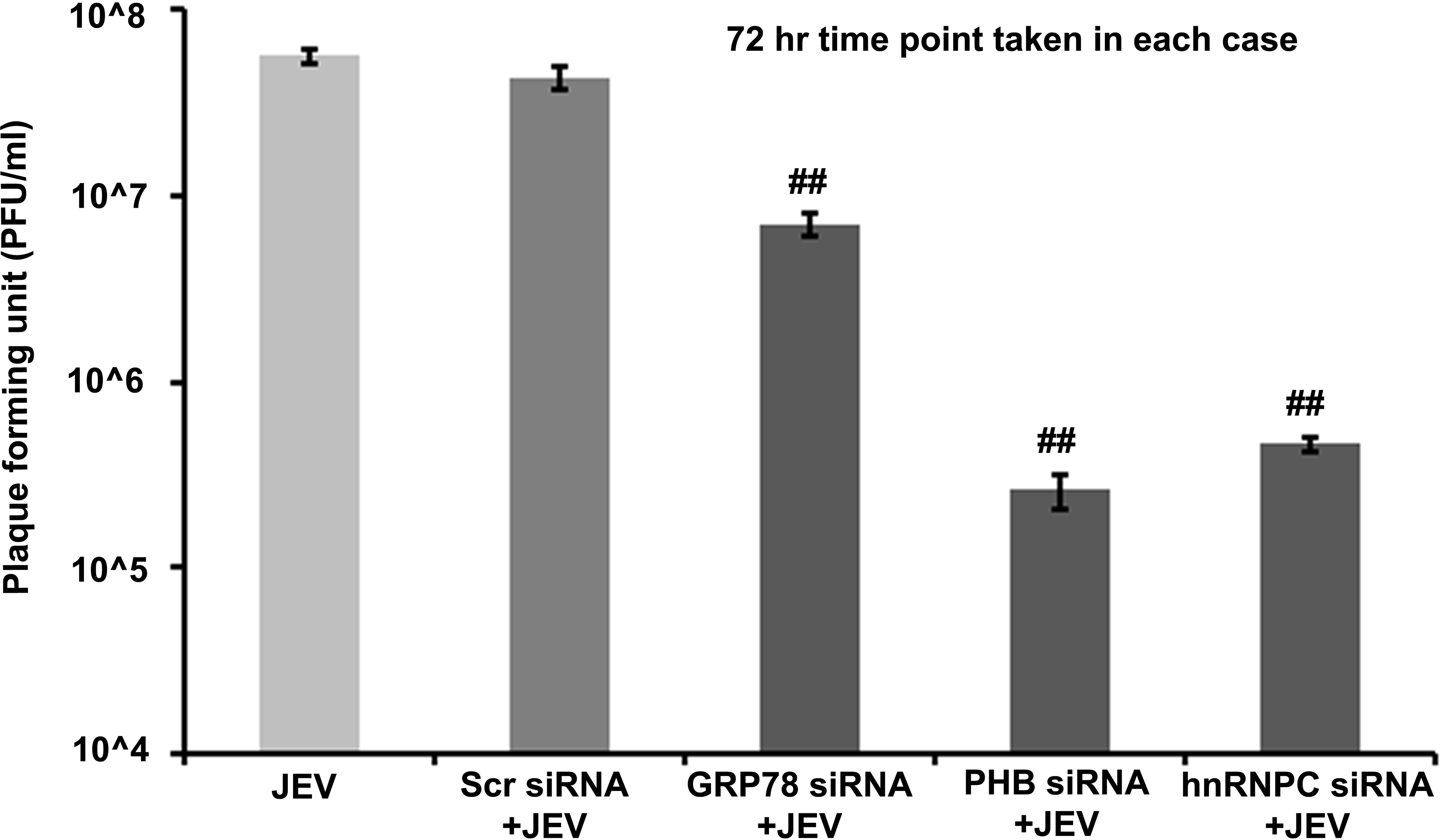

Supplement: Supplementary Figure S7 [file cddis2016394x7.tif]

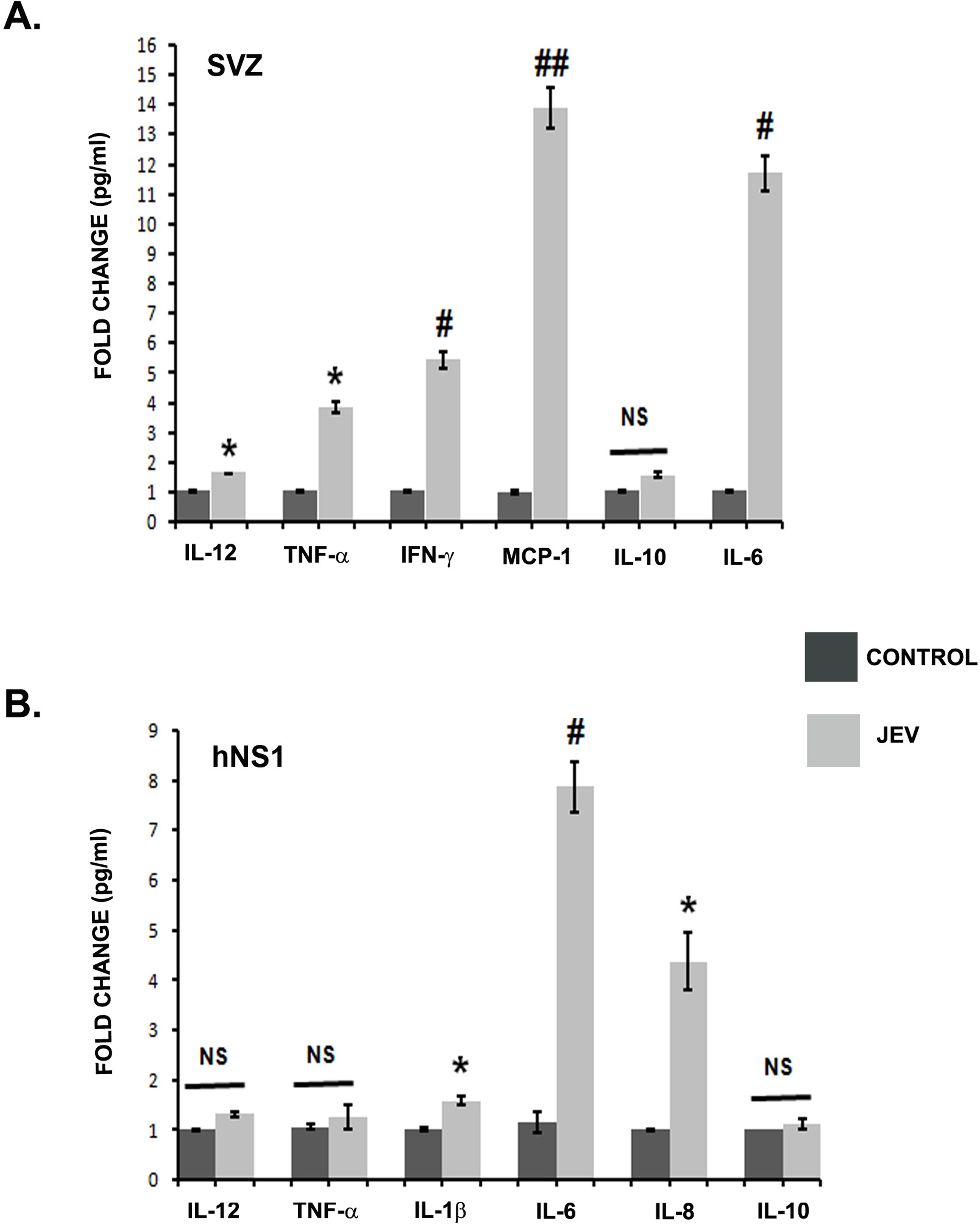

Supplement: Supplementary Figure S8 [file cddis2016394x8.tif]

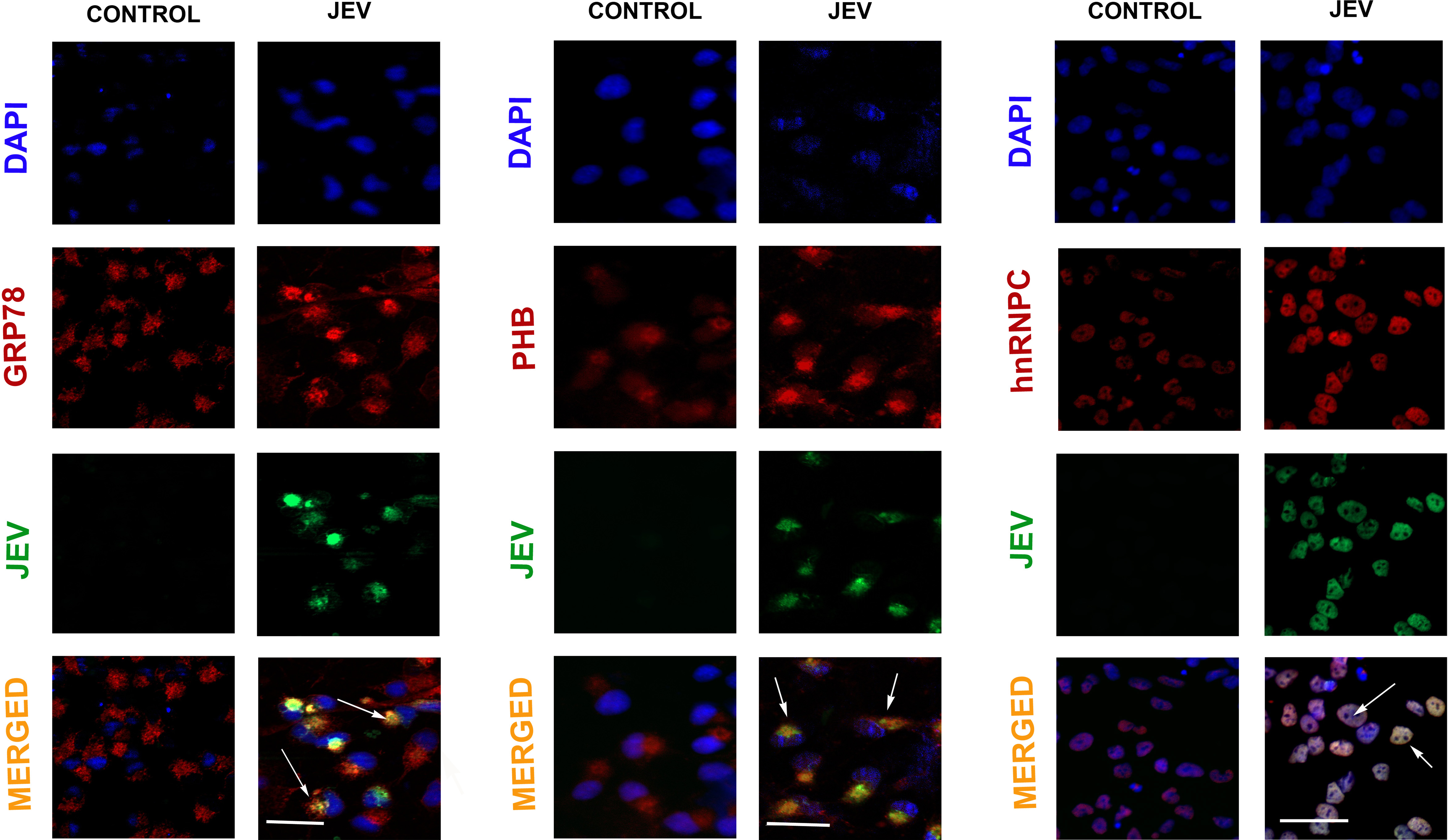

Supplement: Supplementary Figure S9 [file cddis2016394x9.tif]
