# Supplementary material for: Japanese encephalitis virus induces human neural stem/progenitor cell death by elevating GRP78, PHB and hnRNPC through ER stress
Source: Cell Death Dis. 2017 Jan 19;8(1):e2556–. doi: 10.1038/cddis.2016.394 (PMC5386351; doi:10.1038/cddis.2016.394)
Supplement: Supplementary Tables [file cddis2016394x10.docx]

**Table – S1 List of mouse specific primers**

| **Name of Primer (Mouse)** | **Forward / Reverse** | **Sequence(5’ – 3’)** |
| --- | --- | --- |
| **GRP78** | F | ACTTCAATGATGCCCAGCGA |
|  | R | AGCCTTTTCTACCTCACGCC |
| **Calreticulin** | F | GTGTCAGGTTCGGGTGAGAG |
|  | R | GGCCCTTATTGCTGAAGGGT |
| **Vimentin** | F | GAACCTGAGAGAAACTAACC |
|  | R | GATGCTGAGAAGTCTCATTG |
| **hnRNPC** | F | AAAAGTGAACCGAGGAAAAG |
|  | R | TCAAATGAGGAACTGAGTAGAG |
| **Prohibitin** | F | GCTCGATTCGATGCTGGAGA |
|  | R | GCTGGCAGGTAGGTGATGTT |
| **HYOU1** | F | GAAGATTTTGCTGAACAACC |
|  | R | GGAATTGATATCTTTCCGGC |
| **ATF4** | F | CAAGGAGGATGCCTTTTC |
|  | R | GTCATCCATTCGAAACAGAG |
| **ATF6** | F | AGCTGTCTGTGTGATGATAG |
|  | R | GTGATCATAGCTGTAACTGTC |
| **XBP1** | F | GAAGAAGAGAACCACAAACTC |
|  | R | GTGTTCTTAACTCCTGGTTC |
| **PERK** | F | AGGCTTTTCCATCCTCAGCC |
|  | R | CCTCTGGGCTCCTCCTTACT |
| **IRE1** | F | TGTGTCTACCTGGGACGGAA |
|  | R | CCAGCGGAGGACAAGGAAAT |
| **Sec61** | F | GCCGGGCTATCACTGACG |
|  | R | CCAAGCCGTACCCTTTTTGC |
| **Sec62** | F | TTCCAAAAAGGAGGAAACTC |
|  | R | CCACACAAAAACCTCATTTC |
| **Sec63** | F | CACAGCTAATCAGGGAAATTG |
|  | R | GAACTGTTGATCTTCTTCCAG |
| **GAPDH** | F | ATGGCAAGTTCAAAGGCACAGTCA |
|  | R | TGG GGGCATCAGCAGAAG G |

**Table—S2 List of human specific primers**

| **Name of Primer (Human)** | **Forward / Reverse** | **Sequence(5’ – 3’)** |
| --- | --- | --- |
| **GRP78** | F | CTGCTGTTTTCAGATGGAGGT |
|  | R | GCGCTCCTTCAGCTTTTTGT |
| **Calreticulin** | F | CGCTTTTATGCTCTGTCGGC |
|  | R | CTCAGCGTATGCCTCATCGT |
| **Vimentin** | F | CTAACCAACGACAAAGCCCG |
|  | R | TTATCTGCTGGTATATGAGTGCTGC |
| **hnRNPC** | F | TCTTCAGCTACATTTTCGGCT |
|  | R | GGACGGAGAAGGGTGTTCTG |
| **Prohibitin** | F | GAGGTCAGAGTGGAAGCAGG |
|  | R | CACCGCTTCTGTGAACTCCT |
| **HYOU1** | F | CTGGGATTGGACCACGTCAC |
|  | R | GGACGGAGAAGGGTGTTCTG |
| **ATF4** | F | CCTAGGTCTCTTAGATGATTACC |
|  | R | CAAGTCGAACTCCTTCAAATC |
| **ATF6** | F | AATATATGCTAGGGTTAGAGGC |
|  | R | TTCTCTGACACAACTTCATC |
| **XBP1** | F | AGAGTCTGATATCCTGTTGG |
|  | R | AGTTCATTAATGGCTTCCAG |
| **PERK** | F | ATCCCCCATGGAACGACCTG |
|  | R | ACCCGCCAGGGACAAAAATG |
| **IRE1** | F | CACCCTTTTGGCAAGTCCCT |
|  | R | GAACGGGTGTTTGAGCACGTC |
| **Sec61** | F | ACTTAGTGCTCACAAGCCCC |
|  | R | CTCACTTGCGTGCAGTCCTA |
| **Sec62** | F | GATGGTGAAAAGGAAGAATCC |
|  | R | TACACCTCATTTCCATCCAG |
| **Sec63** | F | CATATCATGTGTGTACGCTG |
|  | R | TGAACTTCCAACTTCAATGG |
| **GAPDH** | F | GCAAATTCCATGGCACCGT |
|  | R | TCGCCCCACTGATTTTGG |

**Table S3­­­-- Details of antibodies used in Western-Blot**

| **Antibody** | **Obtained**  **From** | **Molecular**  **Weight (kDa)** | **Catalogue No.** | **Raised In** | **Dilution Used** |
| --- | --- | --- | --- | --- | --- |
| β actin | Sigma | 42 | A3854 | Mouse | 1:10000 |
| GRP78 | Abcam | 76 | ab21685 | Rabbit | 1:2500 |
| hnRNPC(C1/C2) | Abcam | 41 | ab10294 | Mouse | 1:2000 |
| Prohibitin | Abcam | 30 | ab28172 | Rabbit | 1:1000 |
| XIAP | Abcam | 55 | ab21278 | Rabbit | 1:2000 |
| PARP cleaved | Abcam | 25 | ab32064 | Rabbit | 1:2000 |
| Active Caspase 3 | Cell signalling | 17 | 9664L | Rabbit | 1:1000 |
| Caspase 7 | Abcam | 34 | ab2301 | Mouse | 1:1000 |
| Caspase 8 | Abcam | 20 | ab25901 | Rabbit | 1:1000 |
| Caspase 9 | Abcam | 46 | ab25758 | Rabbit | 1:1000 |
| Cytochrome C | Santa Cruz | 12 | sc - 13561 | Mouse | 1:500 |
